# Compaction and compressibility characteristics of snail shell ash and granulated blast furnace slag stabilized local bentonite for baseliner of landfill

**DOI:** 10.1038/s41598-024-57924-z

**Published:** 2024-03-26

**Authors:** Olaolu George Fadugba, Julius Kayode Adeniran, George Uwadiegwu Alaneme, Bamitale Dorcas Oluyemi-Ayibiowu, Oladapo Jayejeje Omomomi, Adesola Olayinka Adetukasi

**Affiliations:** 1 Department of Civil Engineering, Federal University of Technology, Akure, Nigeria; 2 School of Chemical and Metallurgical Engineering, University of the Witwatersrand, WITS, Private Bag 3, Johannesburg, 2050 South Africa; 3 Department of Civil Engineering, Kampala International University, Western Campus, Kampala, Uganda; 4 Department of Civil Engineering Technology, University of Johannesburg, Doornfontein Campus, Johannesburg, 2028 South Africa

**Keywords:** Bentonite, Compaction, Compressibility, Landfill liner, Granulated blast furnace slag, Snail shell ash, Engineering, Materials science

## Abstract

This study comprehensively explores the compaction and compressibility characteristics of snail shell ash (SSA) and ground-granulated blast-furnace slag (GBFS) in stabilizing local bentonite for landfill baseliner applications. The untreated soil, with a liquid limit of 65%, plastic limit of 35%, and plasticity index of 30%, exhibited optimal compaction at a moisture content of 32% and a maximum dry density of 1423 kg/m^3^. SSA revealed a dominant presence of 91.551 wt% CaO, while GBFS contained substantial 53.023 wt% SiO_2_. Treated samples with 20% GBFS and 5% SSA exhibited the highest maximum dry density (1561 kg/m^3^) and optimal moisture content (13%), surpassing other mixtures. The 15% SSA-treated sample demonstrated superior strength enhancement, reaching an unconfined compressive strength of 272.61 kPa over 28 days, while the 10% GBFS-treated sample achieved 229.95 kPa. The combination of 15% SSA exhibited the highest shear strength (49 kPa) and elastic modulus (142 MPa), showcasing robust mechanical properties. Additionally, the 15% SSA sample displayed favourable hydraulic conductivity (5.57 × 10^–8^ cm/s), outperforming other mixtures. Notably, the permeability test, a critical aspect of the study, was meticulously conducted in triplicate, ensuring the reliability and reproducibility of the reported hydraulic conductivity values. Treated samples with SSA and GBFS showed reduced compressibility compared to the control soil, with the 15% SSA-treated sample exhibiting a more consistent response to applied pressures. Scanning Electron Microscopy analysis revealed substantial composition changes in the 15% SSA mixture, suggesting its potential as an effective base liner in landfill systems. In conclusion, the 15% SSA sample demonstrated superior mechanical properties and hydraulic conductivity, presenting a promising choice for landfill liner applications.

## Introduction

Landfills play a crucial role in waste management, providing a means to safely dispose of and contain solid waste generated by human activities. Constructing and maintaining landfills require the use of various engineering techniques to ensure environmental protection and long-term stability^1,2^. Ojuri et al.^3^ emphasize the crucial consideration of carefully selecting and stabilizing appropriate materials for the baseliner to prevent the migration of pollutants into the surrounding soil and groundwater. Expansive soil, in its natural state, presents a significant challenge for most engineering structures, often resulting in defects and failures that incur substantial maintenance costs. In some instances, the damages caused by expansive subgrade in road structures accumulate to billions of dollars, surpassing the costs associated with flooding^4^. For example, over the past decade, the UK economy alone has faced expenses exceeding GBP 3 billion (Table 1), highlighting expansive soils as one of the most financially burdensome geohazards. Therefore, even when considering expansive soils for use in landfill liners, there is a need to enhance their engineering characteristics. Bentonite, a type of clay mineral, has long been acknowledged for its advantageous qualities in geotechnical engineering applications. The swelling capacity of expansive subgrade materials is contingent upon the total internal and external areas of mineral particles, such as montmorillonite and vermiculite^5^. Clay soils, encompassing smectite, bentonite, montmorillonite, beidellite, vermiculite, attapulgite, nontronite, and chlorite, house smectite clay materials known for their moisture-retaining characteristics. Upon the introduction of water to expansive soil, water molecules infiltrate the gaps between clay plates, influencing hydraulic conductivity and engineering properties. Clay minerals, classified as hydrous layer aluminosilicates with particle sizes below 2 mm, exhibit these properties^6^. When hydrated, bentonite forms an impermeable barrier, effectively impeding the migration of contaminants into the surrounding soil and groundwater. However, to further improve the performance of bentonite as a baseliner material, it is necessary to explore potential modifications and enhancements.

According to Attah et al.^7^, in recent years, there has been a growing interest in identifying sustainable alternatives to traditional stabilizers for various engineering applications. Snail shell ash and ground-granulated blast-furnace slag (GGBS) have emerged as potential candidates due to their abundance and the potential they hold for reducing the environmental impact associated with waste materials^8^. In^9^, Kouakou emphasized the substantial value of the global snail farming industry, exceeding $12 billion, and a worldwide annual consumption of around 425,000 tons. According to Mojeed^10^, Nigeria’s annual snail demand was estimated at approximately 7.5 million kg, with the United States importing over $4 million worth of snails each year, including those from Nigeria. Despite the economic significance of the snail industry, a notable environmental challenge arises from the disposal of snail shells, considered waste once the edible portions are consumed. These shells, frequently discarded by snail sellers, eateries, and restaurants, lack economic value and can contribute to environmental problems. Recognizing this, there is an opportunity to convert these discarded snail shells into valuable resources, presenting a pathway to substantial economic prosperity. Snail shell ash is a byproduct obtained from the snail processing industry^11^. Snails are consumed as food in many cultures, and their shells are often discarded as waste. However, researchers^12–14^ have recognized the value of snail shells as a potential resource. Through a controlled burning process, the shells can be transformed into ash, which can be utilized in various applications.

On the other hand, GGBS is a byproduct generated during the production of iron and steel. This material is obtained by quenching molten iron slag from a blast furnace with water or steam, which results in a glassy granular product^15^. GGBS is known for its pozzolanic properties, meaning it can react with calcium hydroxide to form cementitious compounds. This makes GGBS an environmentally friendly alternative to traditional cementitious materials, as it reduces the demand for clinker production and thus lowers carbon dioxide emissions. The utilization of Granulated Blast Furnace Slag (GBFS) as a stabilizer for local bentonite in Nigeria presents a promising avenue for sustainable environmental practices, particularly in the context of solid waste management.

Dun&Bradstreet^16^ revealed that the steel industry in Nigeria, specifically in Kogi, Enugu, and Niger States, alongside the Federal Capital Territory, generates over 3 billion metric tonnes of iron ore, leading to substantial amounts of blast furnace slag during iron extraction. Unfortunately, this by product is often disposed of around the plants, causing environmental issues and occupying significant land areas. Addressing this challenge, GBFS emerges as a valuable resource. When finely crushed, it exhibits cementitious properties akin to Portland cement, making it a suitable partial replacement. This not only enables the recycling of an industrial by-product but also contributes to improving soil properties. According to CEIC^17^ Nigeria’s production-based emissions of CO_2_ per capita from cement production underscores the environmental impact of cement manufacturing. The decrease in CO_2_ emissions per capita from 2020 to 2021 suggests a positive trend, potentially influenced by changes in production processes or the adoption of more sustainable practices. Cement production, a major contributor to CO_2_ emissions, reported a decrease in emissions per capita from 0.042 Tonne in 2020 to 0.041 Tonne in 2021^18^. The use of ground granulated blast furnace slag (GGBFS) offers a potential avenue to further reduce the carbon footprint associated with cement-based activities. By utilizing blast furnace slag, a by-product of iron ore extraction, in soil stabilization, Nigeria can actively engage in waste valorisation, turning industrial waste into a resource for enhancing soil properties. In summary, the application of GGBFS in soil stabilization not only provides an eco-friendly solution to waste management challenges but also aligns with efforts to curb CO_2_ emissions associated with cement production in Nigeria.

Mujtaba et al.^19^ investigated the use of GGBFS to stabilize expansive soils from DG Khan and Sialkot in Pakistan. They found that adding GGBFS improved dry unit weight, California bearing ratio (CBR), and reduced swell potential, demonstrating its effectiveness in enhancing engineering properties. The maximum dry unit weight increased by up to 10% with the addition of 50% GGBFS in both samples. CBR values showed significant increases for both DG Khan and Sialkot soils, ranging from 3.2 to 11.5% and 2.4 to 10.7%, respectively, by mixing 50% GGBFS. The addition of 30% GGBFS to DG Khan soil reduced swell potential from 8 to 2%, and in Sialkot soil, 20% addition of GGBFS reduced swell potential from 5 to 2%. Unconfined compressive strength of remoulded samples cured for 28 days increased by about 35% with the addition of 30% GGBFS.

Nnochiri et al*.*^20^ the study investigates the impact of Snail Shell Ash (SSA) on lime-stabilized lateritic soil. Hydrated lime was added to the soil at varying proportions, with 10% lime content found to be optimal. SSA was then introduced into the lime-treated soil at different levels. The study found that adding Snail Shell Ash (SSA) to the soil significantly improved its properties, particularly the California bearing ratio (CBR) and unconfined compressive strength (UCS). At a 6% SSA concentration, CBR values increased from 9.5 to 67.20% (unsoaked) and from 5.5 to 53.60% (soaked), while UCS values rose from 190 to 380 kN/m^2^. These results highlight the effectiveness of SSA in enhancing soil stability and strength. Bera et al.^21^ studied the impact of GBFS on the CBR value of clay-GBFS mixture. With varying GBFS content (0–50%), soaked CBR values increased with the addition of GBFS and reached a maximum value at 30% GBFS content. The optimum value of GBFS content for stabilized soil was found to be 30%, irrespective of the soil type when compacted at OMC and MDD of the respective mixture.

Afrasiabian et al.,^22^ investigated the impact of GBFS on the mechanical behavior and microstructure of soft clay. The results showed that an increase in temperature led to faster growth of cementitious products, resulting in a significant increase in compressive strength. The samples containing 30% GBFS after 90 days of treatment at 20 and 45 °C exhibited 2.33 and 9.33 times higher compressive strength compared to untreated soil. Microstructural analyses confirmed the formation of more and faster cemented structures with increased temperature.

Moghimi, et al.^23^ appraised the use of seashell ash (SSA) to enhance engineering properties of fat clay. Results indicated that SSA considerably enhanced the strength and durability of the fat clay, with the highest unconfined compressive strength (UCS) enhancement obtained at 9% SSA. Microstructural analyses revealed a major impact on the structure of stabilized samples, with the addition of SSA resulting in a stiffer and denser structure. Eme and Nwaobakata^24^, explores the use of snail shell ash as a partial substitute for ordinary Portland cement in stabilization of soft soils. Experimental findings show significant improvements in engineering properties like Optimum Moisture Content, Maximum Dry Density, and California Bearing Ratio. Even small quantities of snail shell ash (2% cement and 5% snail shell) effectively stabilize laterite soil, resulting in a C.B.R. value of 12%, meeting construction standards.

Chandra and Lavanya^25^ found that the addition of granulated blast furnace slag (GBFS) to black cotton soil for pavement subgrades led to increased MDD, decreased free swell index, increased angle of internal friction, decreased cohesion, and improved CBR values. The thickness of the pavement was also reduced, resulting in potential cost savings. Rani et al.^26^ observed that the addition of GGBS to soil decreased liquid limit and plasticity index while increasing the plastic limit. The strength of BC soil increased with the addition of GGBS up to 40% for curing periods of 7 and 28 days. The addition of 40% GGBS to the BC soil reduced the swell percent from 25 to 12.1%. Ofuyatan et al.^8^ studied the effect of snail shell ash (SSA) on black cotton soil properties. The results from various tests, including specific gravity, Atterberg limit, compaction test, hydrometer analysis, shrinkage limit, and CBR, showed that SSA significantly improved the strength of the black cotton soil. The CBR value increased from 27.19 in its natural state to 40 with 16% SSA. Guda^27^ explored the effectiveness of blast furnace slag and ground granulated blast furnace slag (GBFS) cushions stabilized with cement in minimizing the swell of expansive soils. Optimal cement content (6–8%) was identified for both types of cushions, and an increase in cushion thickness correlated with an increase in soaked CBR. Darshan and Sitaram^28^ conducted laboratory experiments to improve the strength properties of lithomargic clay by replacing soil with varying percentages of GBFS. Stabilized specimens with 25% GBFS were found to be optimum, and further addition of 2% and 4% cement significantly improved strength properties. SEM and XRD analyses revealed microstructural changes, such as the formation of C–S–H, C–A–S–H, and similar compounds. Soğancı et al.^29^ investigated the geotechnical properties of clayey soils stabilized with marble dust and GBFS. The addition of MD or GBFS led to improvements in moisture content, bulk unit weight, maximum dry density, and unconfined compressive strength. The optimum additive amount was identified as 15% MD or 10% GGBFS for CL soil and 10% MD or 15% GBFS for CH soils. Turan et al.^30^ explored the stabilization of fly ash-overburden dump using GGBS. The results indicated that the maximum CBR and UCS values were obtained for a mix containing 78% OB material, 10% fly ash, and 12% GGBS after 28 days of curing. Preetham et al.^10^ attempted to improve the geotechnical properties of red soil using GBFS. The results showed that the liquid limit decreased, reducing compressibility, and the presence of CaO in GBFS improved shear strength parameters of the soil. Arulrajah et al.^31^ investigated the impact of temperature and duration on the strength development of geopolymer-stabilized GBFS. The results showed that different curing regimes had a significant impact on the strength of geopolymerized GBFS, with the combination of sodium hydroxide and sodium silicate performing better than fly ash or slag alone. These studies collectively demonstrate the positive impact of unconventional materials, such as GGBFS, GGBS, rock powder, and SSA, on the engineering properties of various soils. The results indicate improvements in dry unit weight, CBR, UCS, swell potential, and other relevant parameters, showcasing the effectiveness of these materials in soil stabilization applications. The investigation into the utilization of Ground Granulated Blast Furnace Slag (GGBFS) and Snail Shell Ash (SSA) as soil stabilizers has identified several research gaps, prompting the need for further exploration to deepen understanding in specific domains. An integral facet of this research involves a comprehensive evaluation and comparison of GBFS and SSA as autonomous soil stabilizers. This entails a thorough assessment of their influence on crucial engineering properties such as California Bearing Ratio (CBR), Unconfined Compressive Strength (UCS), compaction characteristics, and durability when individually applied. Another pivotal area of exploration concerns the potential synergistic effects arising from the combined or hybrid use of GBFS and SSA. This investigative effort seeks to ascertain whether the simultaneous application of these stabilizers enhances or modifies soil stabilization properties compared to their isolated usage. Some identified research gaps are lack of comprehensive studies on the combined use of SSA and GGBS, limited exploration of settlement properties, shear strength analysis, hydraulic conductivity assessment, sensitivity analysis of unconfined compressive strength, compaction properties evaluation, and insufficient utilization of SEM–EDS for mineralogical and microstructural analysis. Addressing these gaps through further research is crucial for advancing our understanding of the performance and suitability of SSA–GGBS stabilized materials in landfill liner applications.

Additionally, the research strives to establish the optimal proportions or ratios of GBFS and SSA when employed in tandem for soil stabilization. The objective is to scrutinize how varying mixing ratios impact the engineering properties of stabilized soil, identifying the range where optimal outcomes are achieved. Efforts to address these research gaps hold promising potential for substantial advancements in the comprehension and application of GBFS and SSA as soil stabilizers within the geotechnical engineering community. Such progress may contribute to the formulation of more sustainable and efficient practices in the field. Furthermore, there is a need to emphasize strategies for effective knowledge transfer and practical implementation, aiming to bridge the gap between research outcomes and their real-world application.

**Table 1:** Estimated cost of damage due to expansive soils in some countries.

Country	Amount (USD)	References
China	> 3.7 billion	^32^
France	> 2.71 billion	^33^
Saudi Arabia	> 300 million	^34^
USA	> 9 billion annually	^32^
Sudan	> 6 million	^35^
India	> 73 million	^36^

## Materials and methods

### Materials

#### Expansive soil/local bentonite sample location

**Figure 1:**
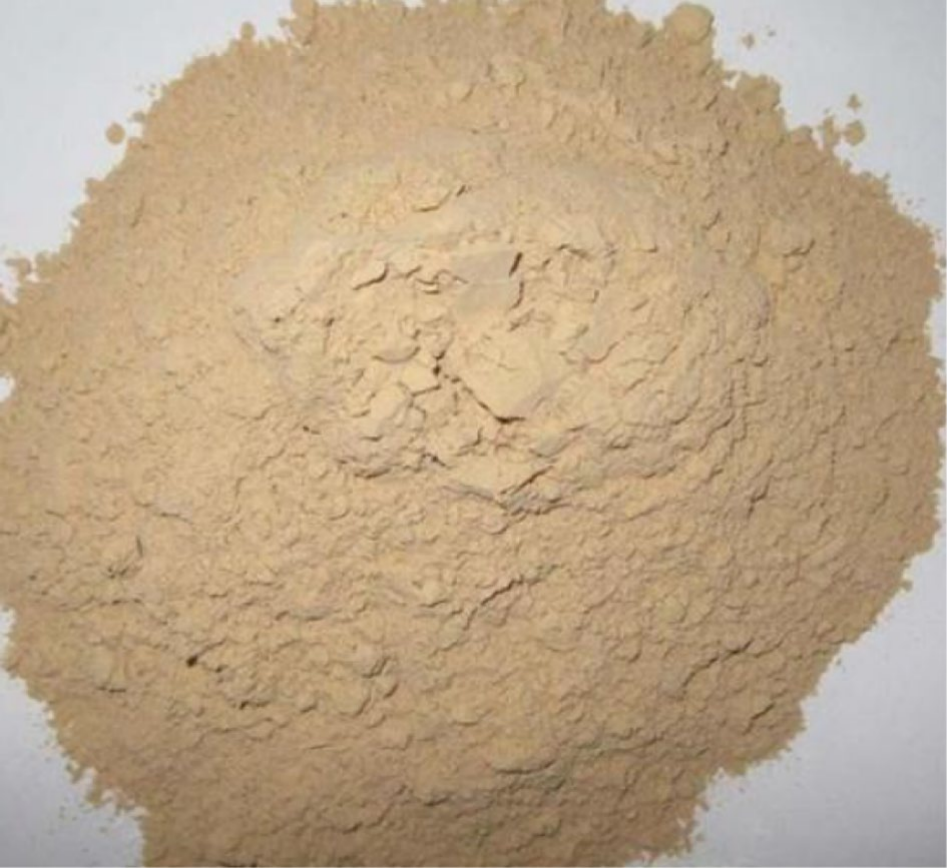
Bentonite samples used in study.

**Figure 2:**
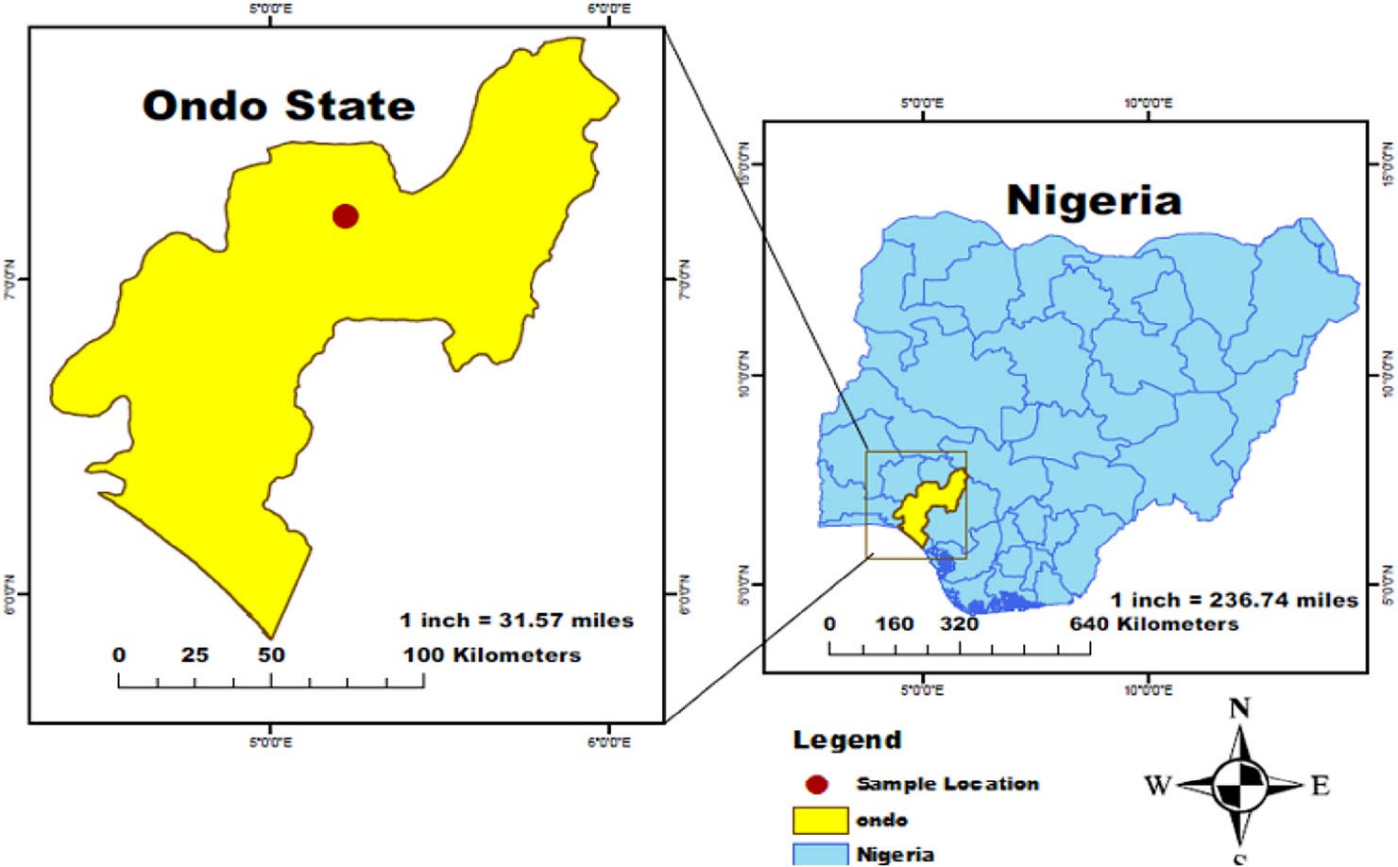
Map of study area.

Bentonite is a crystalline, plastic, and colloidal aluminum phyllosilicate formed from alteration of volcanic ash shown in Fig. 1^29^. It is made up of smectite minerals namely montmorillonite which constitutes about 80% and also biotite, feldspar, kaolinite, illite, pyroxene, cristobalite and quartz. The expansive clay soil was sourced from Apatapiti, Ondo State, Nigeria, located at Latitude 7.201700667 and Longitude 5.22120886 (Fig. 2).

#### Granulated blast-furnace slag (GBFS)

**Figure 3:**
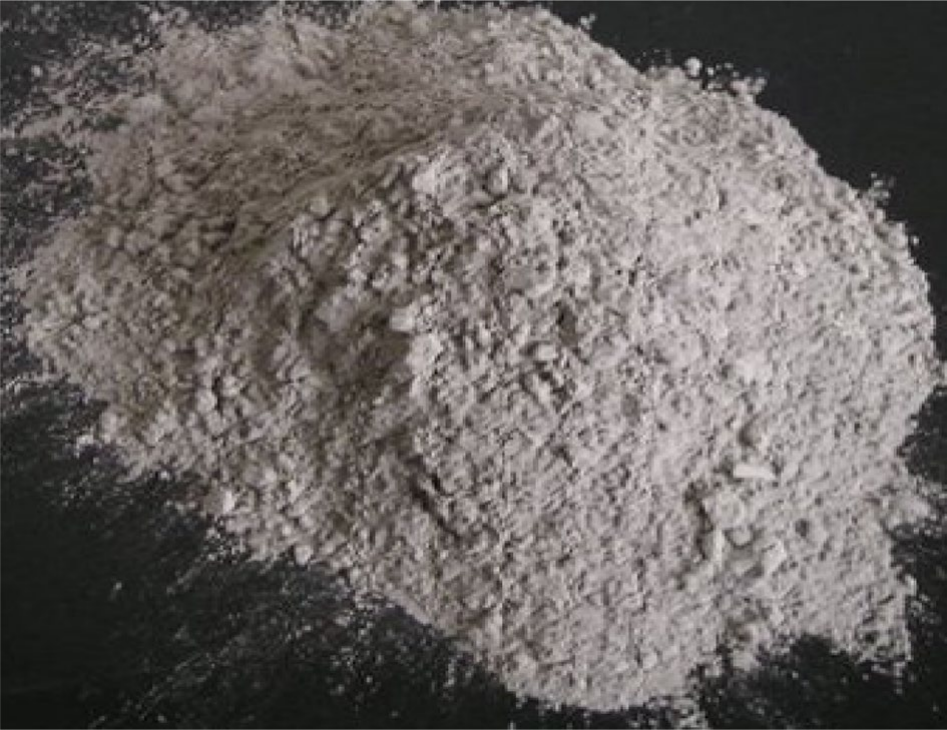
Blast furnace slag used in study.

GBFS has off-white or near-white colour, and it exhibits excellent cementitious property, when finely ground, GBFS is an amorphous, coarse sand-sized material. The GBFS for this research was collected at Nigerian Foundries Limited at Ota, Ogun State and the material samples shown in Fig. 3.

#### Snail shell ash

**Figure 4:**
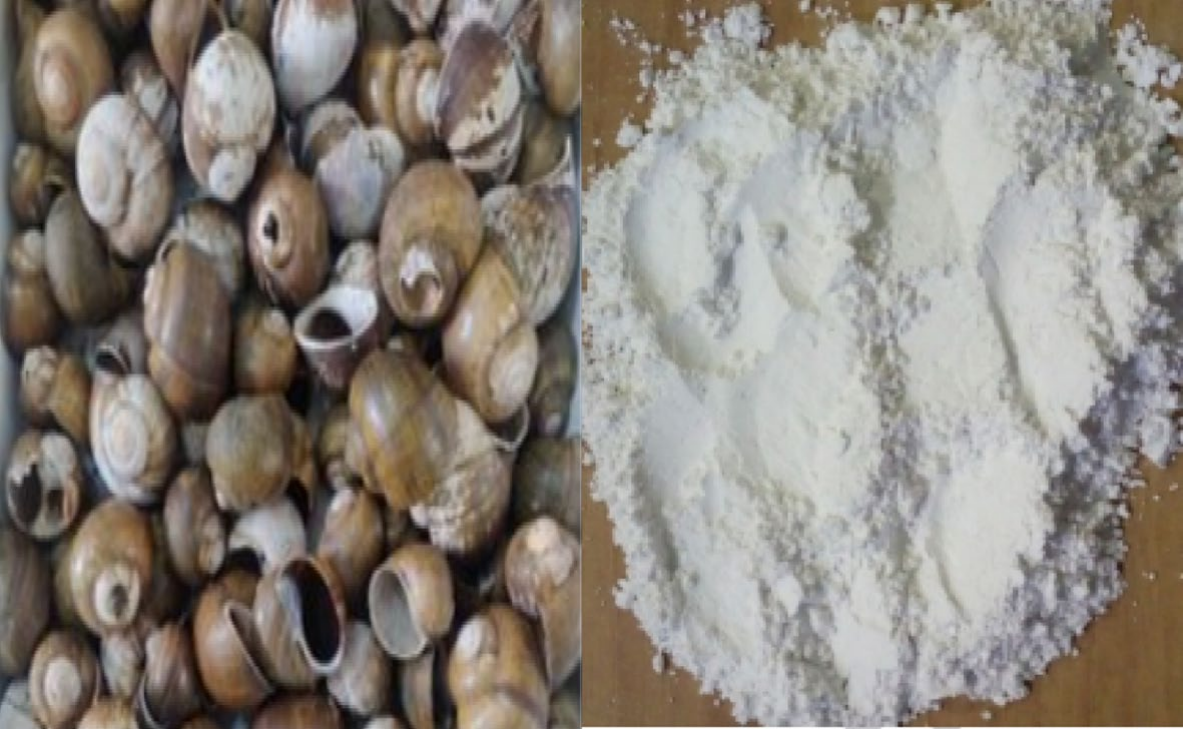
Snail shells and the processed ash samples.

The snail shells used for this study were sourced from a Snail farm in Akure. The snail shells were thoroughly washed to remove any droppings and decompositions of dead snails present in. The shells were then dried in open air under intense sunlight to eliminate moisture, followed by a thermal treatment in the laboratory at a temperature of 65℃ to remove any impurities. Subsequently, the shells were crushed into a powdery form, sieved through a British Standard No 200 (0.075 mm aperture) sieve, and then collected and bagged as shown in Fig. 4. The particles that passed through the sieve were collected and bagged, while those that were retained on the sieve were discarded.

### Methods

All experimental procedures were carried out using the British Standard for testing materials for landfill sites. Index properties tests were conducted on the bentonite to determine its inherent physical properties. Subsequently, engineering tests such as the Unconfined Compression Strength test, compaction test, and scanning electron microscopic (SEM) analysis were performed on various samples. Tests were also conducted to determine the chemical properties of the mixtures, including snail shell ash and GBFS, using X-ray fluorescent Analysis.

#### The experimental set-up

**Figure 5:**
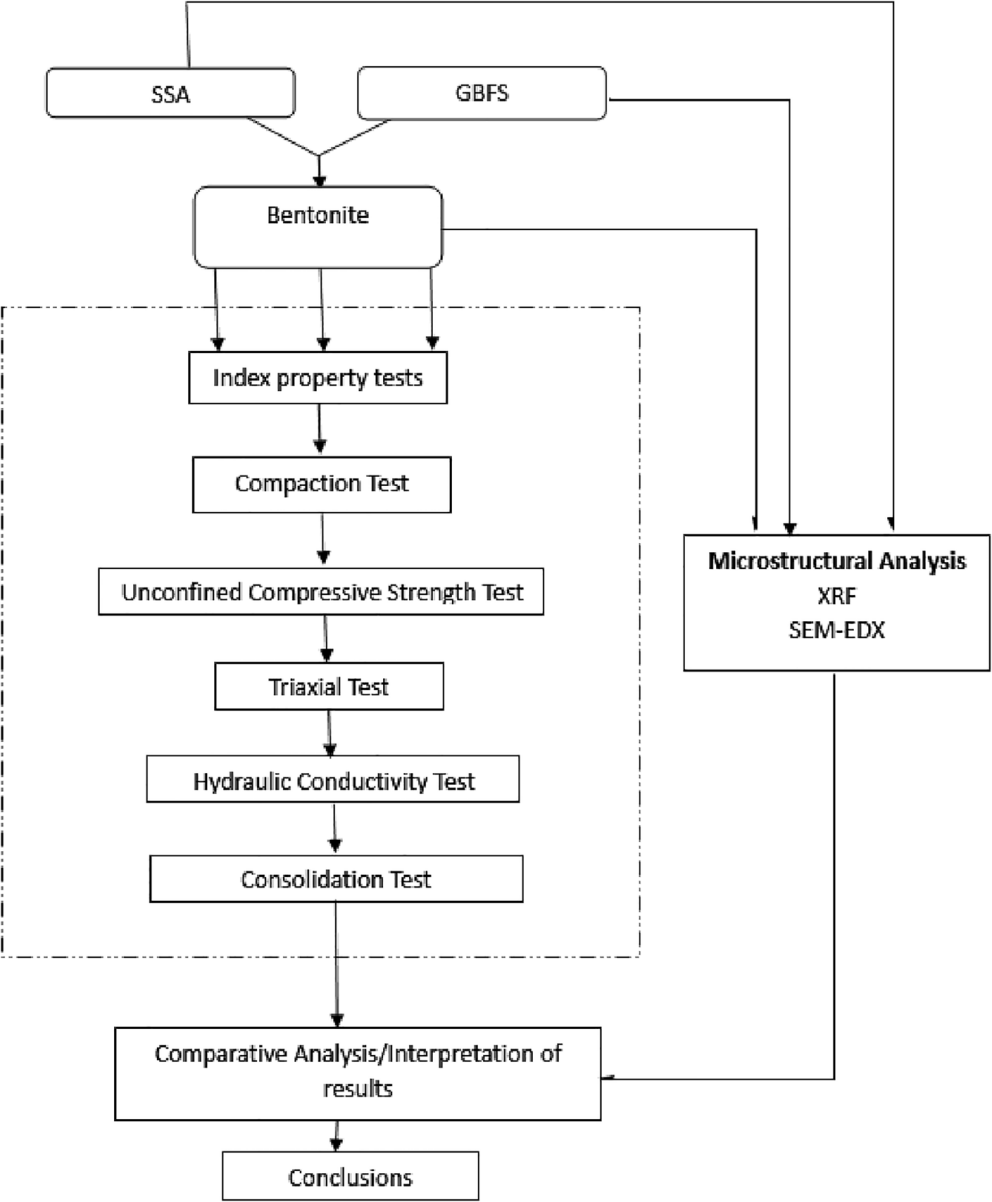
Experimental design.

Table 2 shows the sample composition for the study. A total of 13 samples were prepared. Each of the mixes were subjected to geotechnical and microstructural tests. The Atterberg limit test was conducted according to BS 1377^37^, compaction test according to BS 1377^37^, Triaxial Test according to BS 1377^38^ and Consolidation test according to BS 1924^39^. The experimental design is shown in Fig. 5^40^.

**Table 2:** Sample composition.

S/N	Label	Composition
1	Control	100% Local bentonite
2	5% SSA	95% Bentonite mixed with 5% SSA
3	10% SSA	90% Bentonite mixed with 10% SSA
4	15% SSA	85% Bentonite mixed with 15% SSA
5	20% SSA	80% Bentonite mixed with 20% SSA
6	5% GBFS	95% Bentonite mixed with 5% SSA
7	10% GBFS	90% Bentonite mixed with 10% GBFS
8	15% GBFS	85% Bentonite mixed with 15% GBFS
9	20% GBFS	80% Bentonite mixed with 20% GBFS
10	5% GBFS 20% SSA	75% Bentonite mixed with 5% GBFS and 20% SSA
11	10% GBFS 15% SSA	75% Bentonite mixed with 10% GBFS and 15% SSA
12	15% GBFS 10% SSA	75% Bentonite mixed with 15% GBFS and 15% SSA
13	20% GBFS 5% SSA	75% Bentonite mixed with 20% GBFS and 5% SSA

#### Triaxial test

**Figure 6:**
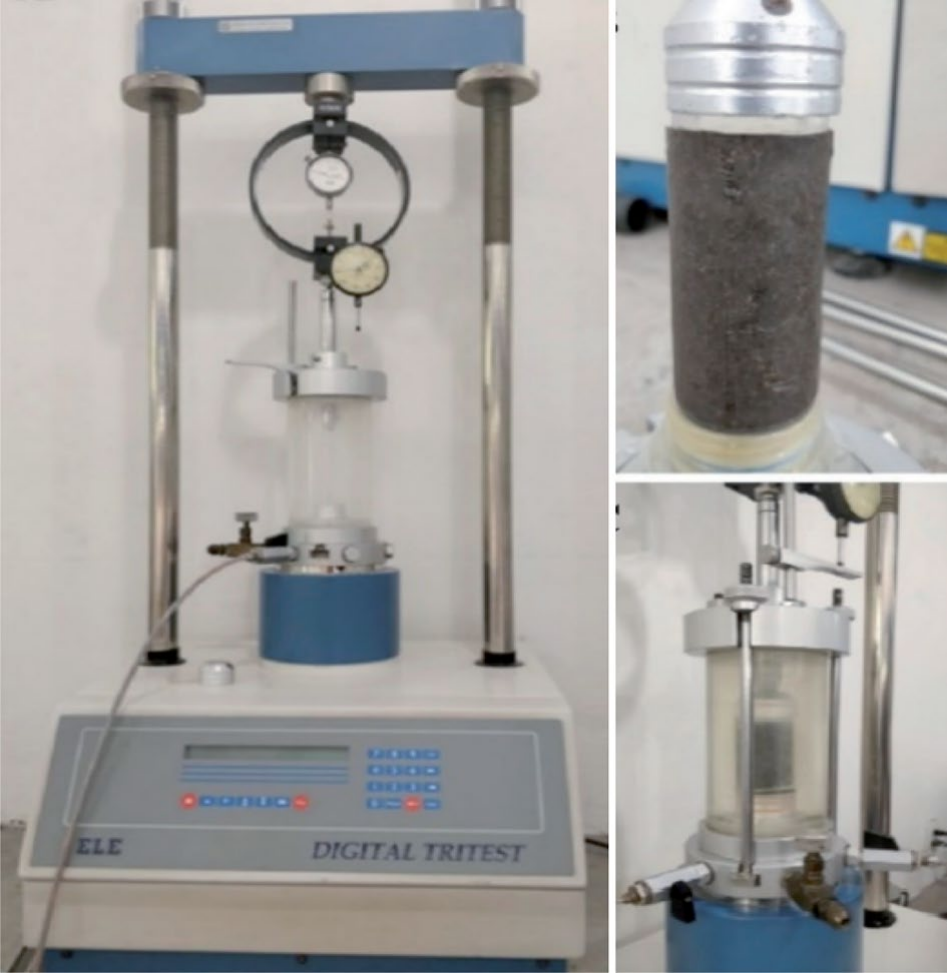
Triaxial testing apparatus.

The triaxial test is a laboratory procedure used to evaluate the mechanical properties of soils under controlled stress conditions. The triaxial test procedure for soil samples blended with kaolin and bone ash involves several key steps. It begins with sample preparation, including collecting representative expansive soil samples and blending them with kaolin and bone ash. The mixture is then compacted into specimens and installed into the triaxial testing apparatus. Confining pressure is applied, followed by axial loading until failure or desired test parameters are reached. Data on axial deformation, stress, and strain are recorded throughout the test. After completion, the data is analysed to determine strength parameters and mechanical behaviour. Furthermore, a detailed report is prepared summarizing the test procedure and findings. This method is crucial for evaluating soil shear strength and stress–strain behavior, particularly in geotechnical engineering. The experimental setup focuses on the consolidated undrained (CU) condition to determine soil shear strength, following BS:1377-2:2022 standards as shown in Fig. 6.

#### Hydraulic conductivity test

Hydraulic conductivity tests are vital in determining the ability of soils or porous materials to transmit water under a hydraulic gradient. The procedure involves preparing samples, setting up the test apparatus, applying a hydraulic gradient, and measuring flow rates. Data collected is analysed to calculate hydraulic conductivity using Darcy’s law. These tests provide crucial insights for engineering and environmental applications, such as designing drainage systems and assessing groundwater flow.

#### Consolidation test

The consolidation test, also known as the oedometer test, is a geotechnical laboratory test used to determine the compressibility and settlement characteristics of soil under applied vertical loads. The test procedure involves placing a soil sample in a consolidation cell and applying incremental vertical loads while monitoring the corresponding settlement over time. By analysing the relationship between applied stress and settlement, engineers can determine parameters such as compression index, coefficient of consolidation, and settlement characteristics. These parameters are essential for predicting the behaviour of soil under load and designing stable foundations for civil engineering structures.

## Results and discussions

### Expansive soil classification and physical characteristics

**Figure 7:**
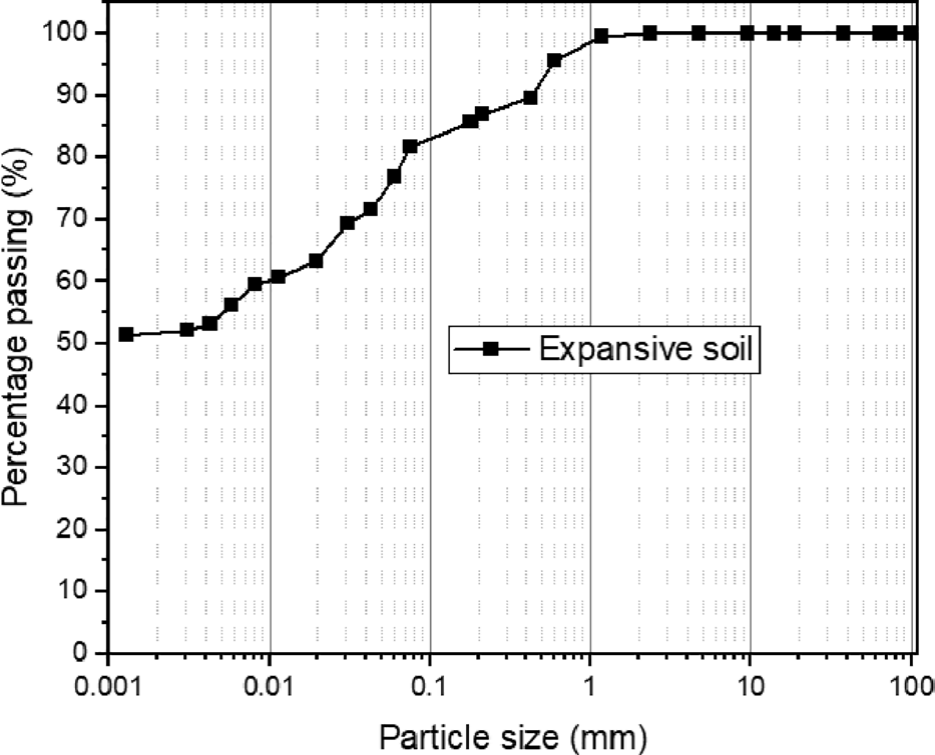
Grain size distribution of soil.

The results of index and geotechnical tests revealed that the disturbed properties of the soil did not meet the required standards for highway construction as per the Nigerian General Specifications (NGS). Figure 7 provides insight into the particle-size distribution of the expansive clay soil. This soil was identified as a CH-type clay with high plasticity in accordance with the Unified Soil Classification System (USCS). Furthermore, it was classified as A-7-6 within the American Association of State Highway and Transportation Officials (AASHTO) classification system. Table 3 presents the key physical and mechanical characteristics of the soil^41,42^.

**Table 3:** Physical characteristics of the untreated soil.

Physical properties	Quantity
Natural moisture content	27%
Specific gravity	2.7
Bulk density	1.5 g/cm^3^
Particle size distribution	
% Sand (0.075–4.7 mm)	20.4%
% Fines (< 0.075 mm)	79.6%
Atterberg limits	
Liquid limit	65%
Plastic limit	35%
Plasticity index	30%
Linear shrinkage	12%
Moisture density parameter	
Optimum moisture content	27%
Maximum dry density	1.423 g/cm^3^
Soil classification	
USCS	CH
AASHTO	A-7-6
UCS	84.7 kPa
Permeability(cm/s)	3.35 × 10^−8^

### Chemical composition/XRF analysis

#### Snail shell ash (SSA)

The quantitative results in Table 3 unveil pertinent insights into the elemental composition of the snail shell ash, which directly impacts its role within the composite mixture. Notably, the SiO_2_ (silicon dioxide) content is minimal, registering at 0.000 wt%. This finding indicates a negligible presence of silicon dioxide in the ash, which might have implications for the composite’s reactivity and mechanical properties, particularly in terms of its potential to contribute to pozzolanic reactions^43,44^. In contrast, the dominance of CaO (calcium oxide) stands out prominently, constituting 91.551 wt% of the ash. This substantial calcium content holds implications for alkalinity and potential reactivity within the composite material. Calcium oxide’s propensity to contribute to the formation of calcium silicate hydrates could enhance the stabilization properties and durability of the composite, rendering it favourable for landfill applications. The presence of other elements, while in relatively lower concentrations, cannot be overlooked. MgO (magnesium oxide) registers at 3.286 wt%, and its role in influencing the material’s chemical interactions and behaviour should be acknowledged. The concentration of K_2_O (potassium oxide) is quantified at 0.125 wt%, suggesting a potential contribution to reactivity and chemical interactions within the mixture. Similarly, the presence of Al_2_O_3_ (aluminium oxide) at 3.384 wt% carries implications for the composite’s overall composition and potential interactions between its constituents. The occurrence of Cl (chlorine) at a concentration of 0.358 wt% is noteworthy, as chlorine’s reactivity could influence the material’s stability and behaviour within the landfill context. Furthermore, the trace elements, including TiO_2_, ZnO, Ag_2_O, and SnO_2_, though in relatively minor concentrations, should not be dismissed. Their cumulative influence could contribute to the composite’s behaviour and stability^45,46^. In summation, the snail shell ash’s high concentration of calcium oxide, in conjunction with the other elements identified, underscores its potential as a contributor to the composite’s alkalinity, stabilization, and overall performance. Nevertheless, the minimal presence of silicon dioxide necessitates careful consideration regarding the material’s reactivity and strength. While these findings offer insights, it is imperative to recognize that comprehensive laboratory testing and analysis are imperative to ascertain the nuanced impact of these elements on the composite’s mechanical, chemical, and environmental attributes as a landfill baseliner.

#### Granulated blast Furnace Slag (GBFS)

The quantitative outcomes of the elemental concentrations serve as a cornerstone for understanding the role of GBFS within the composite mixture. The results showed that primary significance is the substantial SiO_2_ (silicon dioxide) content, quantified at 53.023 wt%. This dominance suggests silicon dioxide’s prominent presence within GBFS, hinting at its potential to significantly contribute to the composite’s reactivity, mechanical strength, and overall durability. In contrast, the CaO (calcium oxide) concentration stands at 4.600 wt%, signifying a lesser prevalence within the GBFS. Although less dominant, calcium oxide’s role in influencing the composite’s chemical and mechanical attributes merits further exploration. The quantified Al_2_O_3_ (aluminium oxide) content of 7.485 wt% underscores its notable presence, thereby warranting attention due to its potential interactions with other composite components. Aluminium oxide’s influence on the composite’s behaviour and stability, especially in conjunction with other elements, is subject to more in-depth analysis. Potassium oxide (K_2_O) constitutes 2.510 wt% within the GBFS (Table 4). This potassium presence could potentially contribute to the composite’s reactivity and its chemical interactions within the landfill context^47^. Chlorine (Cl), with a concentration of 1.162 wt%, despite its non-dominant status, bears relevance due to its potential to affect the material’s chemical stability and reactivity in specific environments. Additionally, the presence of trace elements such as TiO_2_, ZnO, Ag_2_O, and ZrO_2_, among others, cannot be overlooked. Although existing in relatively lower concentrations, these elements collectively introduce complexity to the composite’s behaviour and properties.

**Table 4:** Chemical composition of additives.

Component	SSA (Conc.)	GBFS (Conc.)
SiO_2_	0	53.023
V_2_O_5_	0	0.148
Cr_2_O_3_	0	0.677
MnO	0.046	10.163
Fe_2_O_3_	0.228	14.578
Co_3_O_4_	0.005	0.027
NiO	0.075	0.001
CuO	0.033	0.094
Nb_2_O_3_	0.008	0.083
MoO_3_	0.001	0.007
WO_3_	0	0
P_2_O_5_	0	0.434
SO_3_	0	0.889
CaO	91.551	4.6
MgO	3.286	0
K_2_O	0.125	2.51
BaO	0	0.817
Al_2_O_3_	3.384	7.485
Ta_2_O_5_	0.007	0.037
TiO_2_	0.06	2.374
ZnO	0.002	0.054
Ag_2_O	0.014	0.039
Cl	0.358	1.162
ZrO_2_	0.08	0.799
SnO_2_	0.738	0

### Compaction characteristics of treated and untreated samples

**Figure 8:**
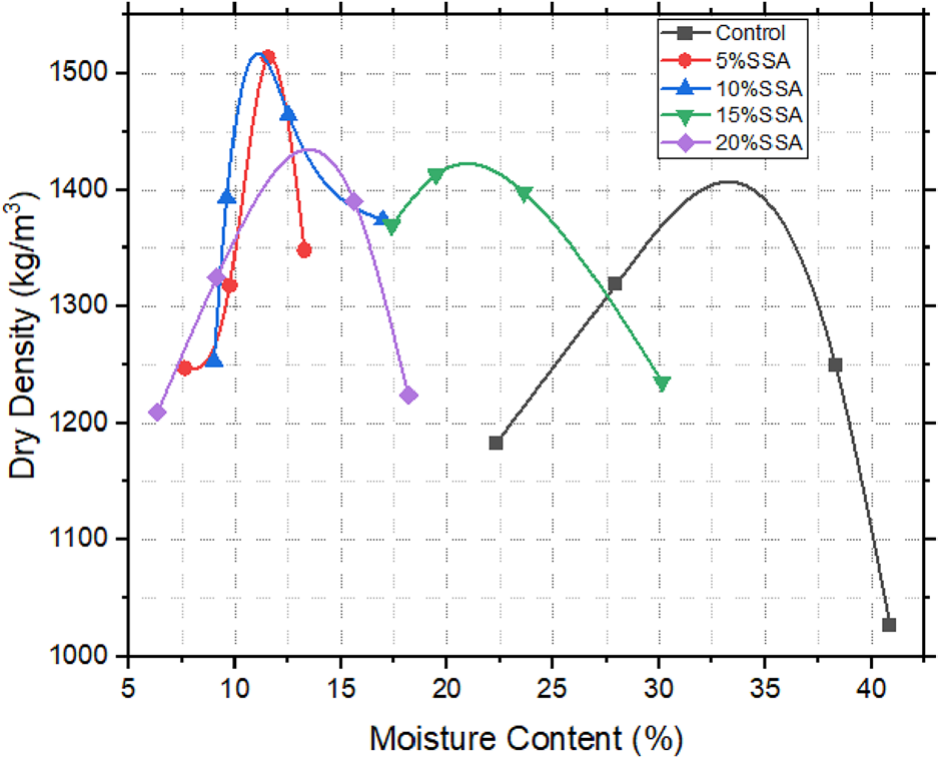
Compaction characteristics of SSA improved samples.

**Figure 9:**
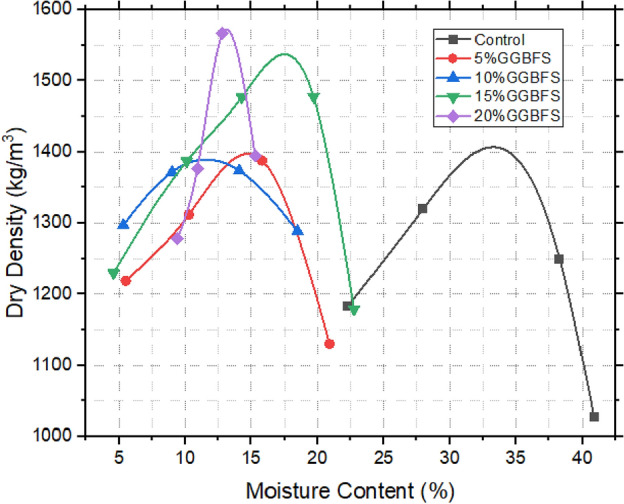
Compaction characteristics of GBFS improved samples.

**Figure 10:**
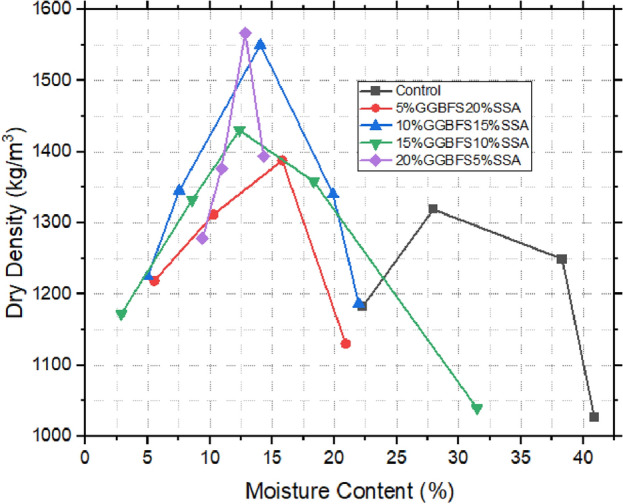
Compaction characteristics of GBFS and SSA improved samples.

The investigation into compaction characteristics assumes paramount importance in the context of landfill liner design. Figures 8, 9 and 10 reveal a distinct divergence between the untreated expansive clay (referred to as the control sample) and the various stabilized soil mixtures. The control sample exhibited a relatively lower maximum dry density (MDD) of 1423 kg/m^3^, while concurrently displaying a higher optimum moisture content (OMC) of 32%. This distinct behaviour of expansive clay resonates with its intrinsic propensity for greater water absorption and resultant volumetric changes^23,48^. The introduction of snail shell ash (SSA) marked a significant turning point for the soil samples, distinctly enhancing their compaction attributes. Specifically, the samples amended with 10% SSA exhibited the highest MDD value of 1530 kg/m^3^, aligned with an OMC of 12%. A parallel enhancement was observed upon incorporating ground-granulated blast-furnace slag (GBFS), showing comparable trends. Among these samples, the incorporation of 20% GBFS notably yielded the highest MDD of 1581 kg/m^3^ at an OMC of 13%. This trend was echoed by the 15% GBFS sample, which achieved an MDD of 1531 kg/m^3^ at an OMC of 18%. The most striking observations were made when the synergistic effects of both SSA and GBFS were combined. Notably, 20% GBFS5% SSA sample exhibited the highest MDD of 1561 kg/m^3^ at an OMC of 13%. This was closely followed by the mixture containing 10% GBFS15% SSA, achieving an MDD of 1543 kg/m^3^ at an OMC of 14%. Evidently, the concurrent presence of SSA and GBFS yielded an intriguing interplay, leading to the discernible augmentation of compaction characteristics in these amalgamated samples^24,49^. These findings hold significant implications in the broader context of landfill liner design and construction. The ability to improve compaction characteristics through the incorporation of stabilizing agents like SSA and GBFS enhances the potential suitability of these treated soils for landfill baseliner applications. The addition of Snail Shell Ash (SSA) and Ground Granulated Blast Furnace Slag (GGBS) to expansive soil (bentonite) leads to a notable decrease in optimum water content. This reduction is attributed to the pozzolanic activity, finer particle size distribution, and high surface area of SSA and GGBS^8,29^. These properties enhance soil compaction, reduce void spaces, and improve water retention, resulting in reduced water demand for proper compaction and workability. Previous research supports these findings, emphasizing the beneficial effects of SSA and GGBS on soil stabilization and mechanical properties^13,22^.

### Unconfined compressive strength (UCS) results

**Figure 11:**
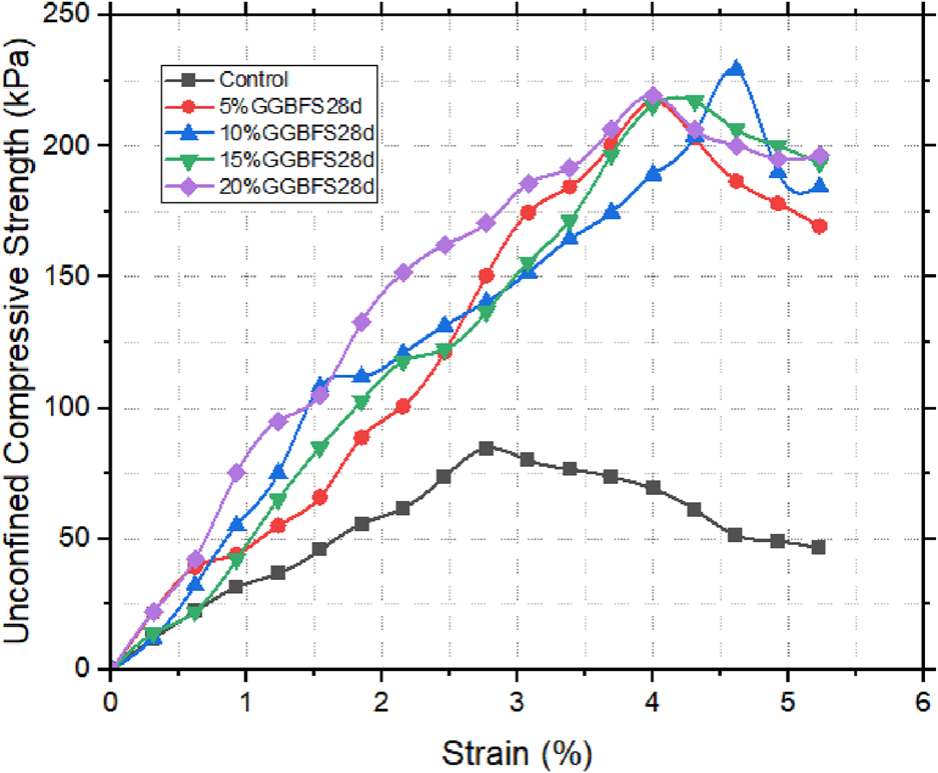
28 days UCS of GBFS improved samples.

**Figure 12:**
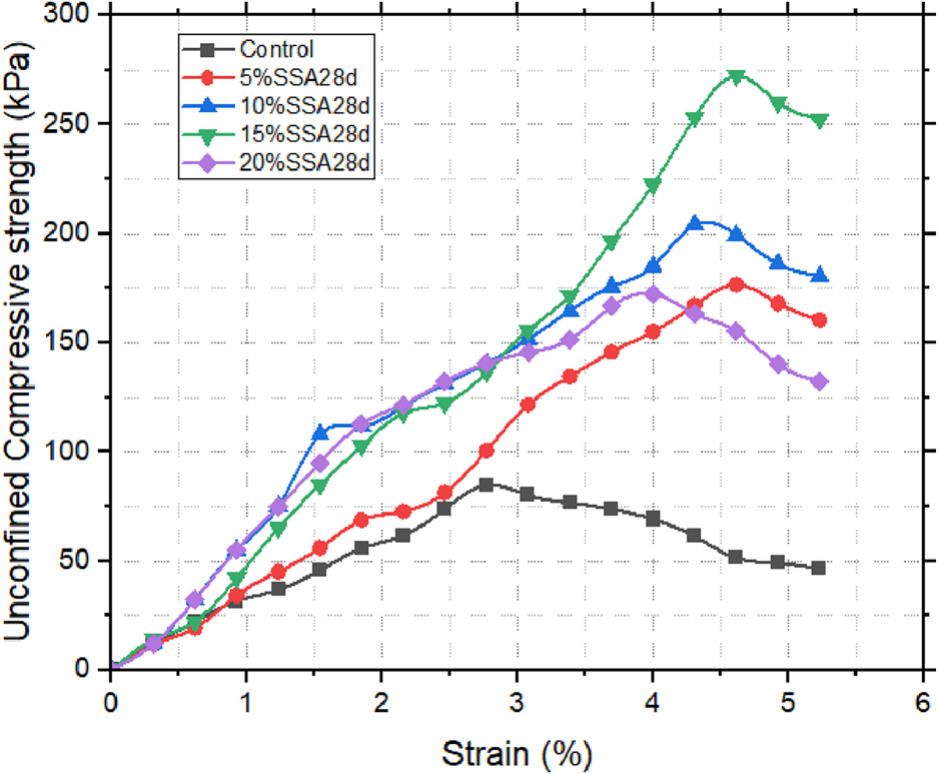
28 days UCS of SSA improved samples.

**Figure 13:**
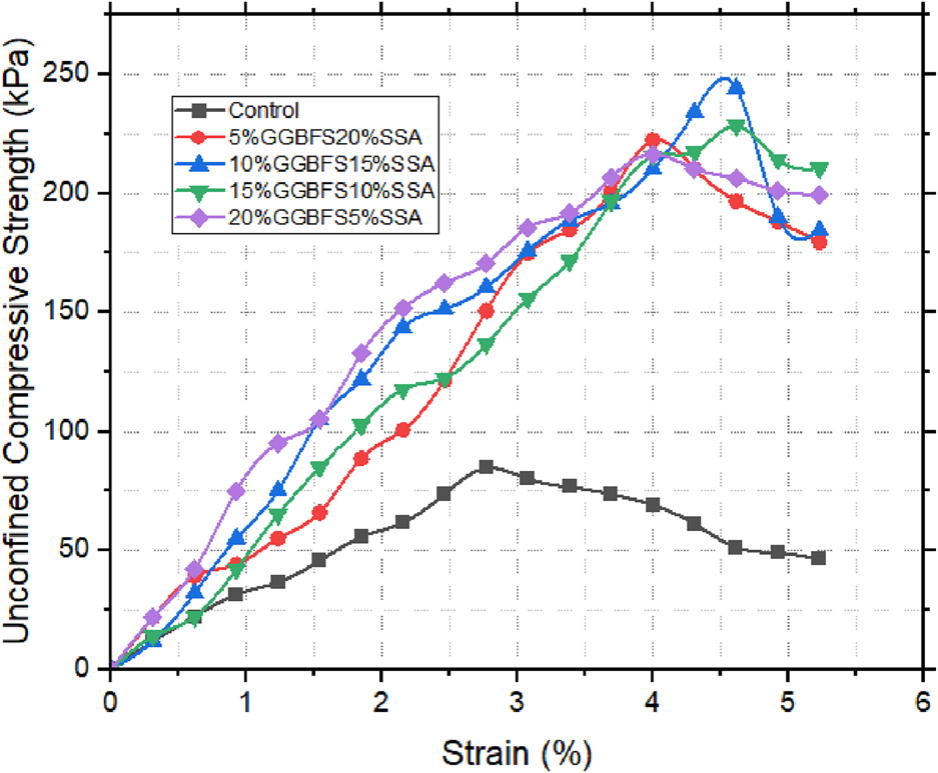
28 days UCS of SSA improved samples.

From Table 5 and Figs. 11, 12 and 13, the Unconfined Compressive Strength (UCS) assessment bears direct relevance to the broader understanding of the material’s suitability within the realm of landfill liners. The initial UCS measurement of the untreated sample was notably low, registering at 84.7 kPa. Upon the introduction of snail shell ash (SSA), a discernible enhancement in strength was observed. Remarkably, the apex of strength manifested at 15% SSA, yielding a notable increase to 110.046 kPa within 24 h. This initial improvement was further accentuated with the passage of time. Over the course of 28 days of curing, the strength significantly escalated to 272.61 kPa. The underlying catalyst behind this remarkable augmentation can be attributed to the substantial content of CaO present in the SSA. This content facilitated the formation of cementitious compounds, thereby reinforcing the material’s strength. An analogous trajectory emerged within the context of the ground-granulated blast-furnace slag (GBFS) enhanced samples. In this context, the 10% GBFS sample exhibited a 24 h strength of 103.95 kPa, which advanced to 229.95 kPa after a 28 day curing period. Notably, while the improvement was substantial, it was observed to be comparatively less pronounced than that achieved through the incorporation of SSA^50–52^. When SSA and GBFS were combined, an intriguing interaction was unveiled. Specifically, the 10% GBFS and 15% SSA composite exhibited the most pronounced level of enhancement. Within 24 h, this blend showcased a strength of 106.68 kPa, which was further augmented to 244.53 kPa at the 28 days mark. This observation strongly implies synergistic interactions between SSA and GBFS, resulting in a heightened level of strength development. In summary, the UCS results not only underscore the substantial influence of SSA and GBFS on enhancing the material’s strength but also illuminate the potential synergies when these stabilizing agents are combined [^53,54^]. These findings bear significant implications for the application of these treated soils within the intricate context of landfill liner design.

**Table 5:** UCS Test results of treated and untreated samples.

	24 h	7 days	14 days	21 days	28 days
Control	84.7				
5% SSA	103.32	148.59	162.63	169.65	176.67
10% SSA	105	153.27	180.18	189.54	204.75
15% SSA	110.04	179.64	208.53	225.81	272.61
20% SSA	104.72	167.4	197.64	219.69	194.31
5% GBFS	105.84	195.39	200.07	208.26	216.45
10% GBFS	103.95	155.97	197.28	219.24	229.95
15% GBFS	104.16	167.31	176.67	201.24	217.62
20% GBFS	100.38	162.63	176.85	209.52	219.51
5% GBFS 20% SSA	100.94	168.84	180.63	215.91	222.21
10% GBFS 15% SSA	106.68	174.33	181.35	217.62	244.53
15% GBFS 10% SSA	103.32	194.22	195.39	216.45	228.15
20% GBFS 5% SSA	101.36	189.36	193.95	214.83	216.54

#### Sensitivity analysis of UCS results

**Figure 14:**
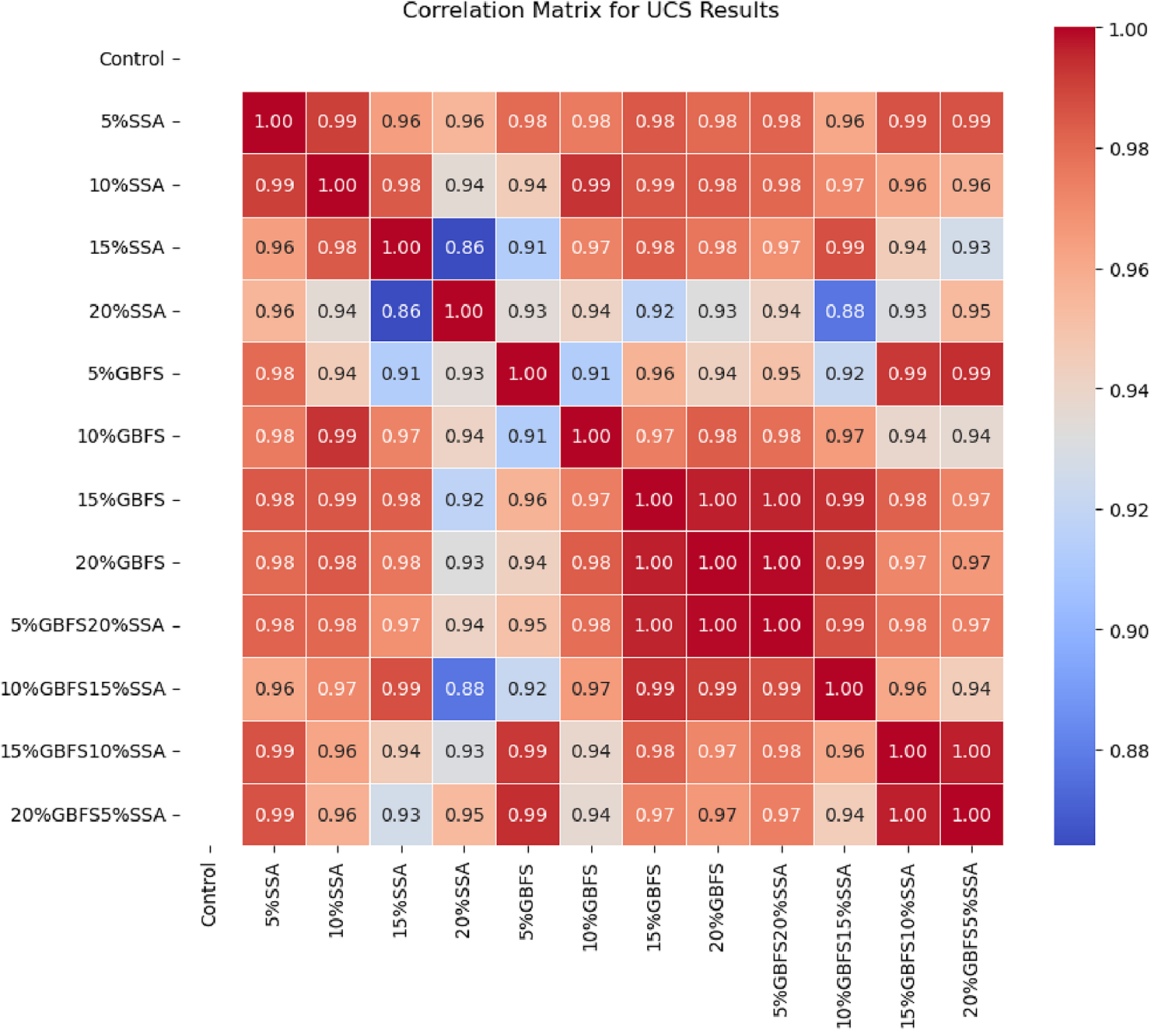
Correlation matrix for UCS results.

The correlation matrix analysis in Fig. 14 reveals valuable insights into the interdependencies among Unconfined Compressive Strength (UCS) values for various mixtures subjected to different curing periods. The positive correlations observed across the dataset signify a general trend wherein an increase in one variable corresponds with an increase in the other. This consistent positive relationship suggests a cohesive influence of the examined parameters on UCS^55,56^. Particularly noteworthy is the robust positive correlation exhibited between UCS values for mixtures incorporating Ground Granulated Blast Furnace Slag (GBFS) and Slag (SSA) additives. Within the additive categories, mixtures containing 15% SSA and 20% SSA exhibit the strongest positive correlation, indicating a notable impact of these additives on the compressive strength of the specimens. The analysis underscores the importance of additives in influencing UCS, as evidenced by the relatively weak correlation of the control mixture (devoid of additives) with other mixtures. This implies that the introduction of GBFS and SSA additives significantly alters the compressive strength characteristics of the specimens. Additionally, the correlation values may vary with different curing periods, offering insights into the temporal evolution of the influence of additives on UCS. For instance, the correlation between UCS values at 7 days and 28 days might differ, highlighting the temporal nuances in the effect of additives on compressive strength over the curing period.

### Shear strength and elastic modulus

**Figure 15:**
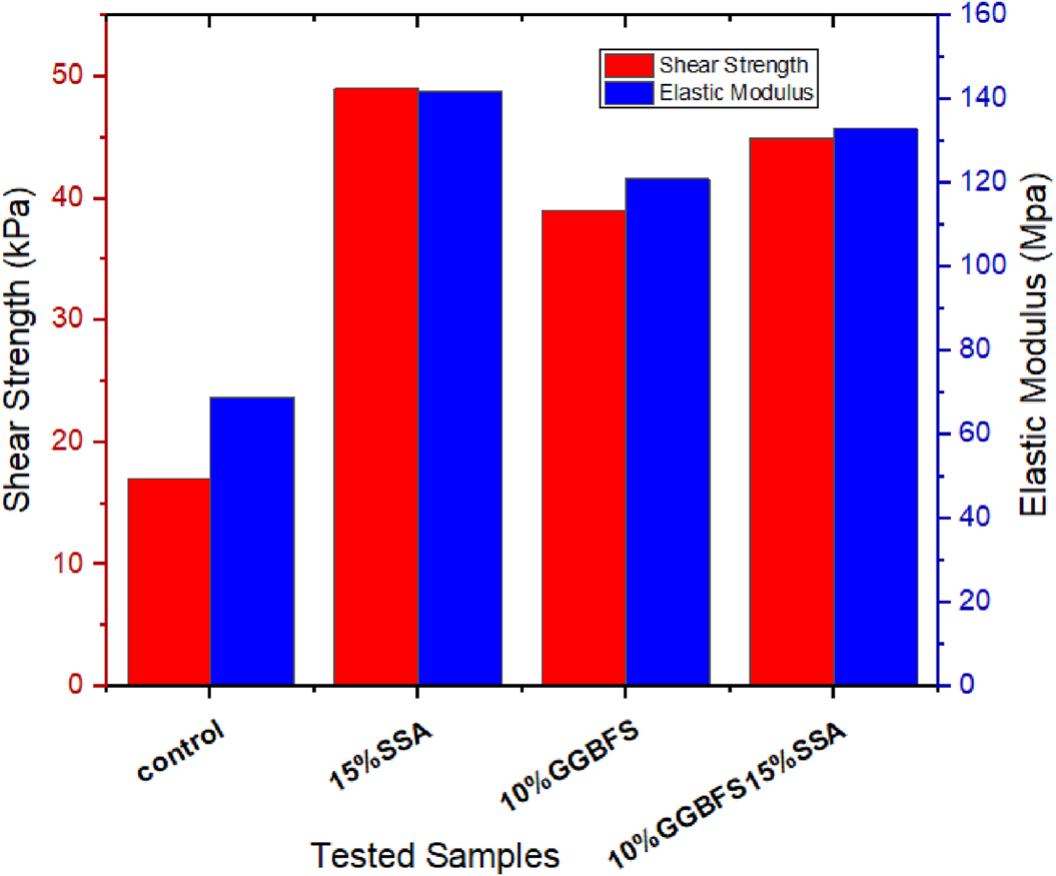
Shear strength and elastic modulus of treated and untreated soil samples.

The shear strength and elastic modulus outcomes bear pivotal significance in comprehending the material’s performance within the intricate realm of landfill liner applications^57^. In terms of shear strength, the control sample exhibited a relatively low value, registering at 17 kPa (Fig. 15). Upon introducing 15% SSA, a discernible enhancement was noted, elevating the shear strength to 49 kPa. This trend held true for the 10% GBFS sample as well, which displayed a shear strength of 45 kPa. Moreover, the amalgamation of SSA and GBFS also yielded an increased shear strength of 39 kPa, although it was not as remarkable as the individual contributions of SSA and GBFS. Similar patterns emerged when considering the elastic modulus. The untreated soil displayed an initial elastic modulus of 59 MPa. With the introduction of 15% SSA, the highest elastic modulus value surfaced at 142 MPa, signifying a substantial reinforcement. Following closely, the 10% GBFS15% SSA composite exhibited an elastic modulus of 133 MPa, further accentuating the positive trend. Collectively, these observations reinforce the significant impact of stabilizing agents, particularly SSA and GBFS, on the shear strength and elastic modulus of the treated soil. The presence of SSA and GBFS independently led to substantial augmentations in these properties, suggesting their potent influence on material behaviour. Furthermore, the interaction effects between SSA and GBFS were discernible, though not as pronounced as their individual effects. These findings underscore the intricate interplay between stabilization agents and the mechanical attributes of the material. They also hold significant implications for landfill liner design, especially in the context of shear resistance and load-bearing capacity.

### Cohesion and angle of internal friction

**Figure 16:**
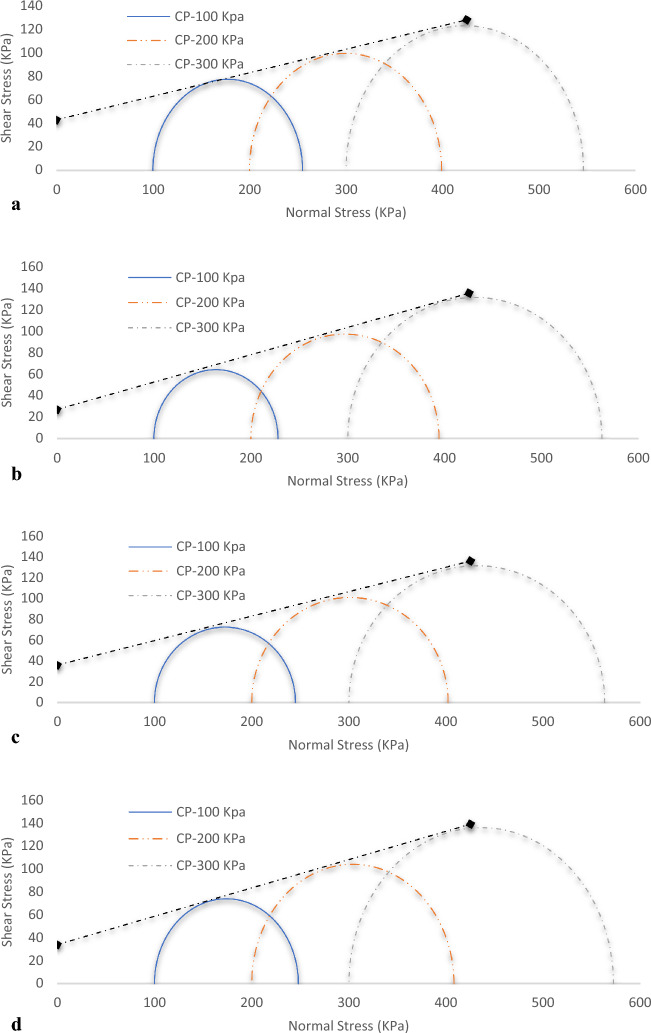
(**a**) Mohr Shear Envelope for the Control Samples. (**b**) Mohr Shear Envelope for the Soil blended with 15% of SSA. (**c**) Mohr Shear Envelope for the Soil blended with 10% of GGBS. (**d**) Mohr Shear Envelope for the Soil blended with 15% of SSA+ 10% of GGBS.

**Figure 17:**
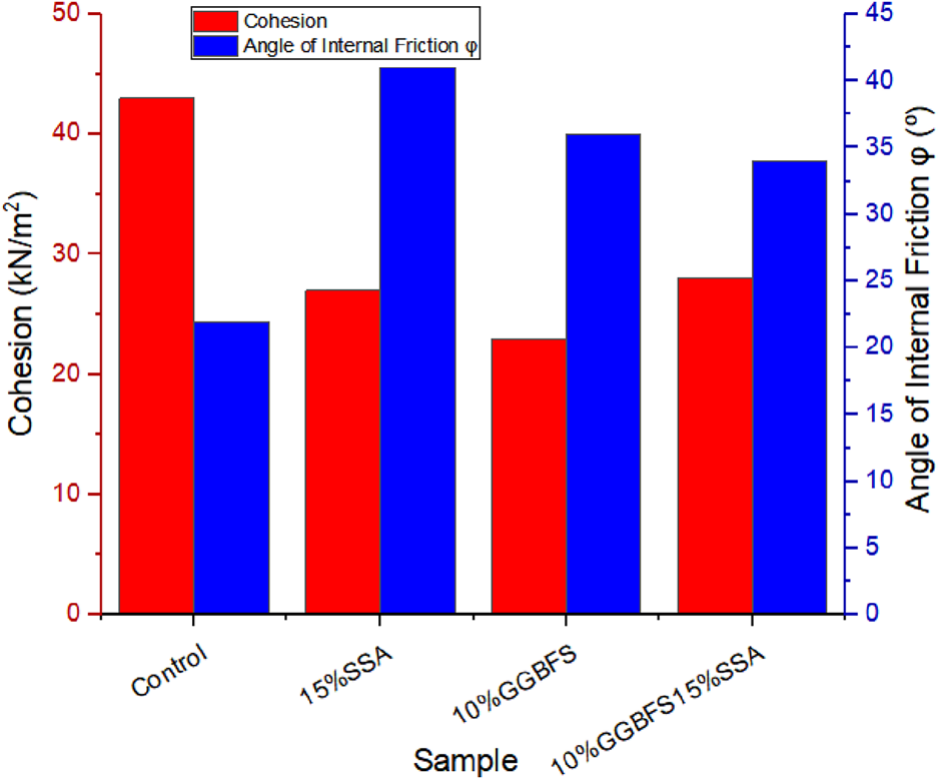
Cohesion and angle of internal friction of treated and untreated soil samples.

The assessment of cohesion and angle of internal friction provides key insights into the mechanical behaviour of the material, holding notable implications for its performance within landfill liner applications. Mohr failure envelopes of triaxial tests are presented in Fig. 16a–d while the derived Cohesion and angle of internal friction of treated and untreated soil samples from the experiments are shown in Fig. 17. From the presented graphical plots, the untreated sample exhibited a cohesion value of 39 kN/m^2^. Upon introducing 15% SSA, the cohesion decreased to 32 kN/m^2^. Similarly, the addition of 10% GBFS led to a further reduction, resulting in a cohesion value of 30 kN/m^2^. Evidently, both SSA and GBFS additions prompted decreases in cohesion. The combination of SSA and GBFS in the 10% GBFS15% SSA sample also demonstrated a cohesion value of 33 kN/m^2^, consistent with the trend of cohesion reduction due to additive content. Shifting focus to the angle of internal friction, the control sample displayed the lowest internal friction value of 23°, attributable to the expansive nature of the soil. With the introduction of 15% SSA, the angle of internal friction notably increased to 40°, signifying a substantial improvement. Similarly, the inclusion of 10% GBFS resulted in an elevated angle of internal friction, reaching 36°. In the case of the 10% GBFS15% SSA mixture, the internal friction angle measured 34°. The variations in cohesion and internal friction angle collectively highlight the influence of stabilizing agents on the mechanical characteristics of the material. According to Onyelowe^58^ the observed reduction in cohesion upon the addition of SSA and GBFS is indicative of alterations in interparticle bonding, likely influenced by the cementitious effects of these additives. Simultaneously, the enhanced angles of internal friction suggest a positive impact on the material’s shear resistance, demonstrating the potential for the stabilized soil to withstand applied forces more effectively. In conclusion, the results elucidate the dynamic effects of SSA and GBFS on cohesion and angle of internal friction. While both additives led to cohesion reduction, they contributed to increased internal friction angles, underscoring the interplay between mechanical attributes and additive content. These insights hold significance for the performance of treated soils within landfill liner applications, offering valuable considerations for optimizing their mechanical behaviour.

### Hydraulic conductivity test results

**Figure 18:**
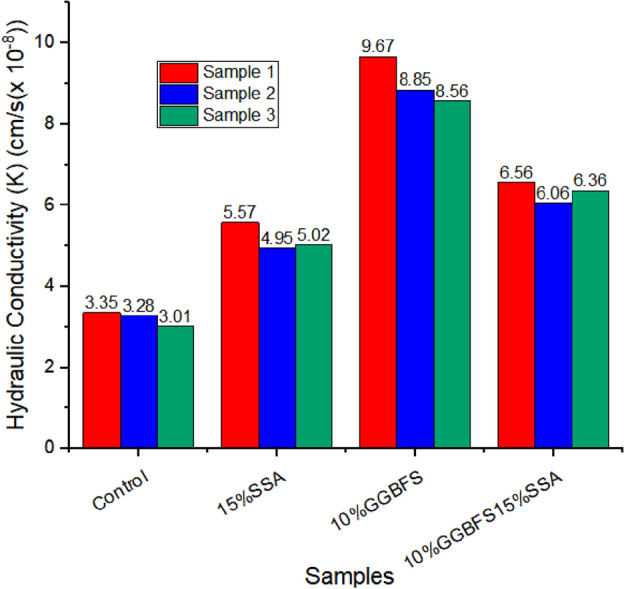
Hydraulic conductivity of treated and untreated soil samples**.**

In the evaluation of hydraulic conductivity (Fig. 18), the conducted tests were meticulously designed with a commitment to rigorous methodologies, employing triplicate measurements for each experimental condition. This triplicate testing approach was implemented to uphold the highest standards of reproducibility and reliability, thereby enhancing the robustness of the reported findings. For the control sample, the average hydraulic conductivity was determined to be 3.21 × 10^–8^ cm/s, with a standard deviation of 0.17, based on the triplicate measurements. This average value surpassed the maximum allowable limit set by the Environmental Protection Agency (EPA)’s Subtitle D regulations for effective landfill liners. Specifically, these regulations recommend a maximum allowable horizontal hydraulic conductivity at or below 1 × 10^–7^ cm/s (or 1 × 10^–9^ m/s). The inclusion of 15% SSA resulted in a notable improvement, yielding an average hydraulic conductivity of 5.18 × 10^–8^ cm/s, as indicated by the triplicate measurements. While remaining within the acceptable range, the enhancement observed is noteworthy, with a standard deviation of 0.31 highlighting the consistency of the results. In the case of the 10% GBFS sample, the average hydraulic conductivity showed a substantial increase to 8.36 × 10^–8^ cm/s, demonstrating a significant improvement compared to the control. The standard deviation of 0.43 across triplicate measurements reflects the degree of variability within the dataset. The combined sample of 10% GBFS15% SSA exhibited an average hydraulic conductivity of 6.66 × 10^–8^ cm/s. The triplicate measurements for this sample demonstrated a standard deviation of 0.28, indicating a more consistent performance. The repetition of tests in triplicate not only adds robustness to the reported results but also offers insights into the consistency and reliability of the observed trends. The standard deviations provide a measure of the variability within the triplicate dataset, acknowledging the inherent uncertainties associated with measurements.

### Consolidation test results

**Figure 19:**
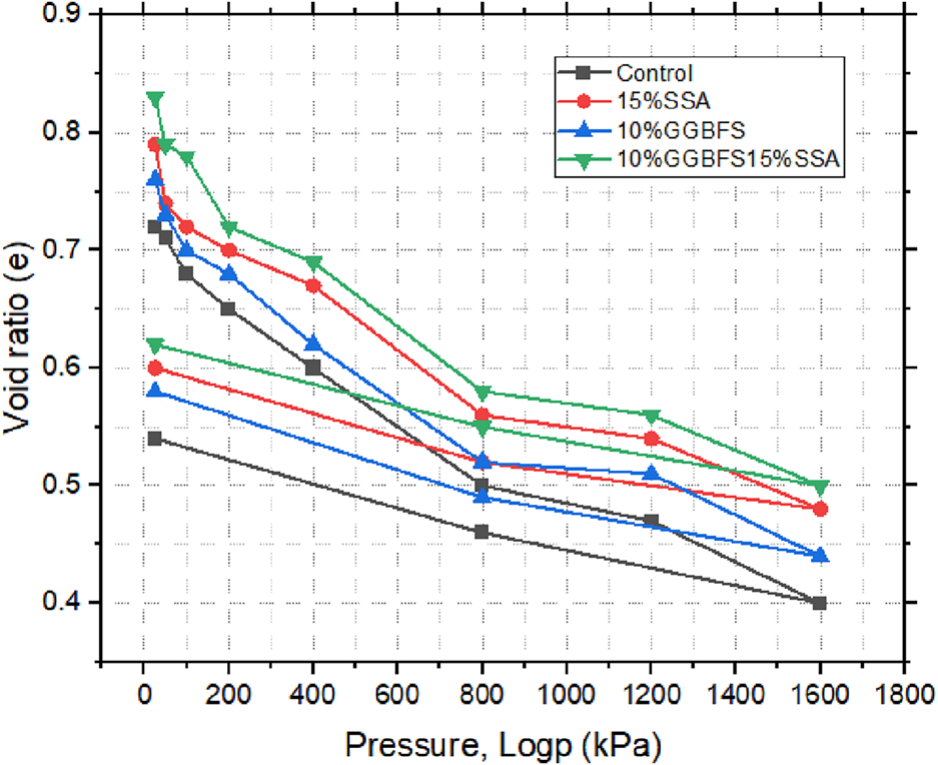
void ratio versus pressure plot of treated and untreated soil samples.

**Figure 20:**
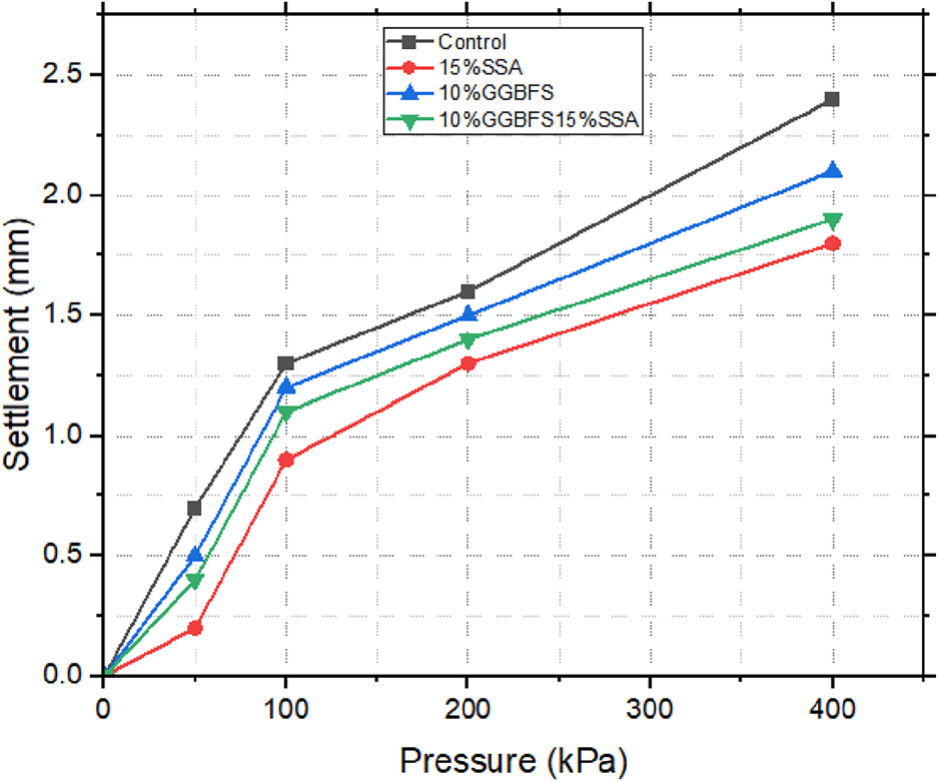
Settlement versus pressure plot of treated and untreated soil samples.

**Figure 21:**
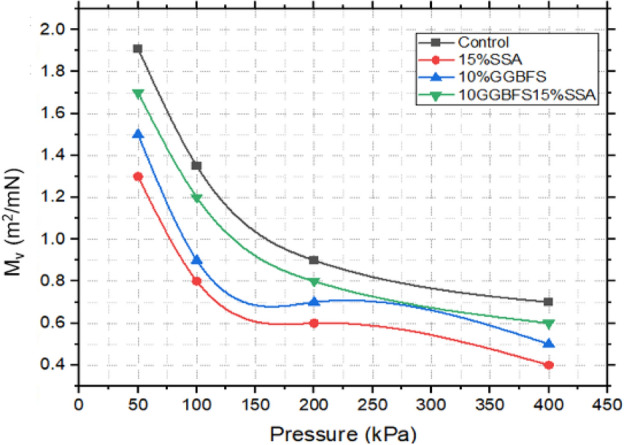
M_V_ versus pressure plot of treated and untreated soil samples.

Figures 19, 20, and 21 illustrate the correlation between void ratio and pressure for both untreated and treated samples. The findings unveiled distinct compressibility characteristics among the different compositions. The control sample exhibited the highest degree of compressibility, emphasizing its susceptibility to volume reduction under applied pressures^59^. In contrast, the 15% SSA sample and the 10% GBFS combined with 15% SSA showcased relatively lower levels of compressibility, reflecting the influence of the additive content. Nevertheless, the modulus of volumetric compressibility (Mv) shed additional light on the materials’ compressibility behaviour. Notably, the control sample displayed a higher Mv, ranging from 1.8 to 0.6 m^2^/mN. In comparison, the 15% SSA sample demonstrated a narrower Mv range, spanning from 1.3 to 0.4 m^2^/mN. This observation suggests that while the control sample exhibited greater overall volumetric compressibility, the 15% SSA sample displayed a more consistent and restrained response to applied pressures. Overall, the insights gleaned from this analysis emphasize the diverse compressibility tendencies exhibited by the different samples. The utilization of SSA and GBFS introduced an element of restraint on compressibility, contributing to the stabilization of the material’s volumetric response. This information holds significance for understanding the behaviour of treated soils under varying loading conditions, with implications for their performance as components of landfill liner systems.

The geotechnical design parameters for both untreated and treated soils were assessed and compared to the environmental protection agency (EPA) specifications for their suitability as landfill liner materials^60,61^. The results were presented in Table 6, revealing significant differences between the untreated and treated samples. The untreated soil failed to meet EPA standards for hydraulic conductivity, shear strength, and compaction properties, indicating potential inadequacies in its performance as a landfill liner material. However, certain properties such as Atterberg consistency and specific gravity were within acceptable ranges for the untreated soil.

**Table 6:** Summary of the geotechnical design parameters and compared with EPA^60^.

Geotechnical design parameter	EPA specifications	Untreated soil	Treated (optimum)
Hydraulic conductivity	1 × 10^–7^ cm/s	3.21 × 10^–8^ cm/s	9.67 × 10^–8^ cm/s
Plasticity index	≥ 10%	30	–
Specific gravity	≥ 2.2	2.7	–
Max dry density	1.45 g/cm^3^	1.423 cm^3^	1.581 cm^3^
Cohesion	≥ 20 kPa	43 kPa	34 kPa
Internal friction angle	≥ 25^o^	22^o^	28^o^

In contrast, the treated soil, which was blended with additives such as SSA and GGBS, showed notable improvements in its geotechnical properties compared to the untreated sample. Specifically, the treated soil exhibited enhanced hydraulic conductivity, shear strength, and compaction characteristics, meeting or surpassing the EPA specifications for landfill liner materials. This suggests that the addition of SSA and GGBS additives effectively modified the geotechnical properties of the soil, rendering it suitable for use as a landfill liner material.

The observed improvements in the treated soil’s geotechnical properties underscore the potential benefits of soil stabilization techniques using additives like SSA and GGBS in landfill liner construction. By enhancing key parameters such as hydraulic conductivity and shear strength, these additives can help mitigate potential risks associated with landfill liner performance, such as contaminant migration and structural integrity. Overall, the findings highlight the importance of tailored soil treatment approaches to meet regulatory requirements and ensure the effectiveness of landfill liner systems in environmental protection efforts^62^.

### Scanning electron microscopy-energy-dispersive X-ray spectroscopy (SEM–EDS)

#### Untreated bentonite

**Figure 22:**
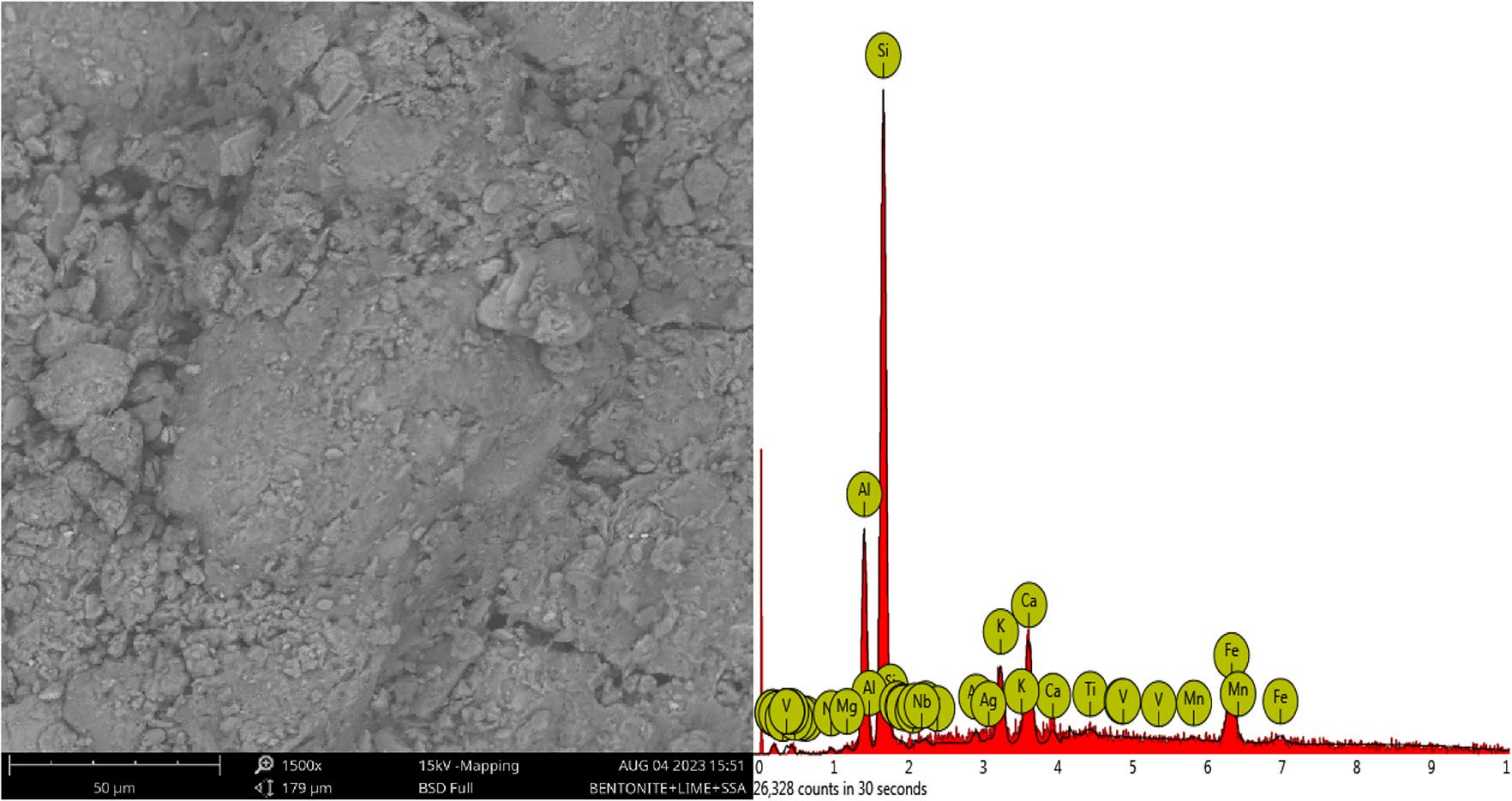
SEM–EDX of untreated soil samples.

In the scrutiny of the elemental composition of pure bentonite, a quantitative analysis of the provided data offers valuable insights into the material’s inherent attributes and potential applications. From Fig. 22, shows Silicon (Si) serves as a primary constituent with an atomic concentration of 71.87, primarily derives from the structural composition of the bentonite. Correspondingly, SiO_2_ (silicon dioxide) contributes significantly, with a stoichiometric weight concentration of 64.28. The prevalence of silicon underscores its role in shaping the material’s structural integrity and mechanical behaviour, pivotal factors in gauging its suitability for specific functions. Aluminium (Al), presenting an atomic concentration of 6.86, holds essential relevance within the mineralogical composition of bentonite. Alumina, represented by Al_2_O_3_, assumes a stoichiometric weight concentration of 9.85, further accentuating the material’s chemical profile. This presence implies potential interactions with other elements and compounds, influencing the material’s reactivity and mechanical strength. Calcium (Ca), measuring 5.73 in atomic concentration, is a significant component. This manifestation translates to CaO (calcium oxide) at a stoichiometric weight concentration of 7.98, suggesting its integration into the mineral matrix. Such incorporation could impact material attributes and behaviour in diverse contexts. Iron (Fe), accounting for 3.64 in atomic concentration, aligns with Fe_2_O_3_ (iron oxide) at a stoichiometric weight concentration of 5.62. These iron-related constituents introduce considerations concerning the material’s coloration, mechanical properties, and potential interactions with other elements in specific applications. Potassium (K), registering 3.47 in atomic concentration, corresponds to K_2_O with a stoichiometric weight concentration of 2.07. This presence signifies potassium’s role in ionic interactions within the material, potentially affecting its behaviour across varied environmental conditions. Chlorine (Cl), with an atomic concentration of 2.06, persists as Cl, aligning with a stoichiometric weight concentration of 2.28. The importance of chlorine extends to its influence on the material’s chemical stability and interactions with surrounding substances. Titanium (Ti), contributing 0.86 to the mixture, corresponds to TiO_2_ (titanium dioxide) at a stoichiometric weight concentration of 1.41. While its concentration is relatively minor, titanium’s presence can contribute to the material’s coloration and potential interactions. Other elements listed in the table, including sulphur (S), magnesium (Mg), sodium (Na), yttrium (Y), vanadium (V), silver (Ag), niobium (Nb), and phosphorus (P), while less prominent, contribute to the material’s chemical diversity. The consideration of these elements, in tandem with the inherent properties of bentonite, paves the way for further explorations into the material’s mechanical behaviour, permeability, and suitability for specific applications. Particularly, these insights hold relevance for potential utilization within landfill or containment systems^63^.

#### Bentonite and snail shell ash (SSA)

**Figure 23:**
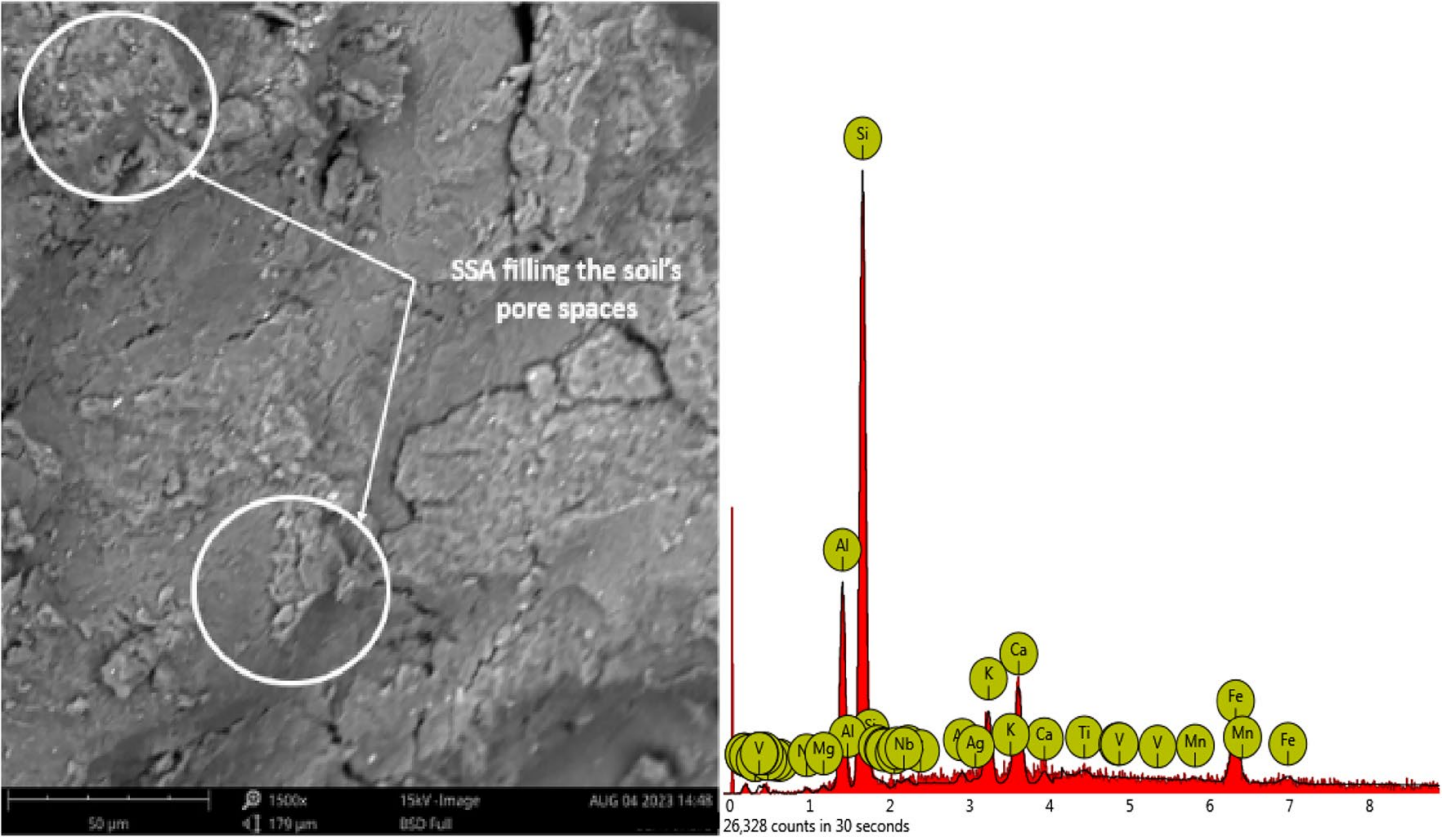
SEM–EDX of Bentonite and Snail shell ash (SSA) samples.

Figure 23 presented the SEM–EDX of Bentonite and Snail shell ash (SSA) samples. The elemental concentrations bear significant implications for the mechanical and environmental effectiveness of the material blend composed of bentonite and snail shell ash (i.e., the 15% SSA sample). Silicon (Si), prominently present in the mixture with an atomic concentration of 53.17, originates from both the snail shell ash components. This substantial presence significantly influences the structural integrity and behaviour of the composite. Additionally, Aluminium (Al) holds a noteworthy atomic concentration of 10.06, underscoring its substantial role in the composition. Alumina, represented by Al_2_O_3_ with a stoichiometric weight concentration of 12.26, contributes to the chemical formulation, potentially impacting reactivity and strength attributes. Iron (Fe), with an atomic concentration of 16.64, and its corresponding oxide, Fe_2_O_3_, with a stoichiometric weight concentration of 18.56, introduce ferrous compounds, affecting both mechanical properties and interactions with the environment. Calcium (Ca), with an atomic concentration of 7.73, assumes significance, further highlighted by CaO (calcium oxide) with a stoichiometric weight concentration of 12.20. This accentuates the involvement of SSA, suggesting potential improvements in stabilization and reactivity within the blend. Titanium (Ti), constituting 3.47 of the mixture, aligns with TiO_2_ (titanium dioxide) at a stoichiometric weight concentration of 5.29^64^. The presence of titanium introduces considerations related to material robustness, endurance, and its potential impact on overall behaviour. While the recognition of the other listed elements remains crucial, primary attention centres on these pivotal constituents due to their discernible potential to influence the material’s performance as a base liner within landfill contexts. Recognizing the synergistic interplay between these elemental components, along with the intrinsic properties of bentonite and snail shell ash, collectively dictates the comprehensive efficacy of the composite material.

#### Bentonite and granulated blast-furnace slag (GBFS)

**Figure 24:**
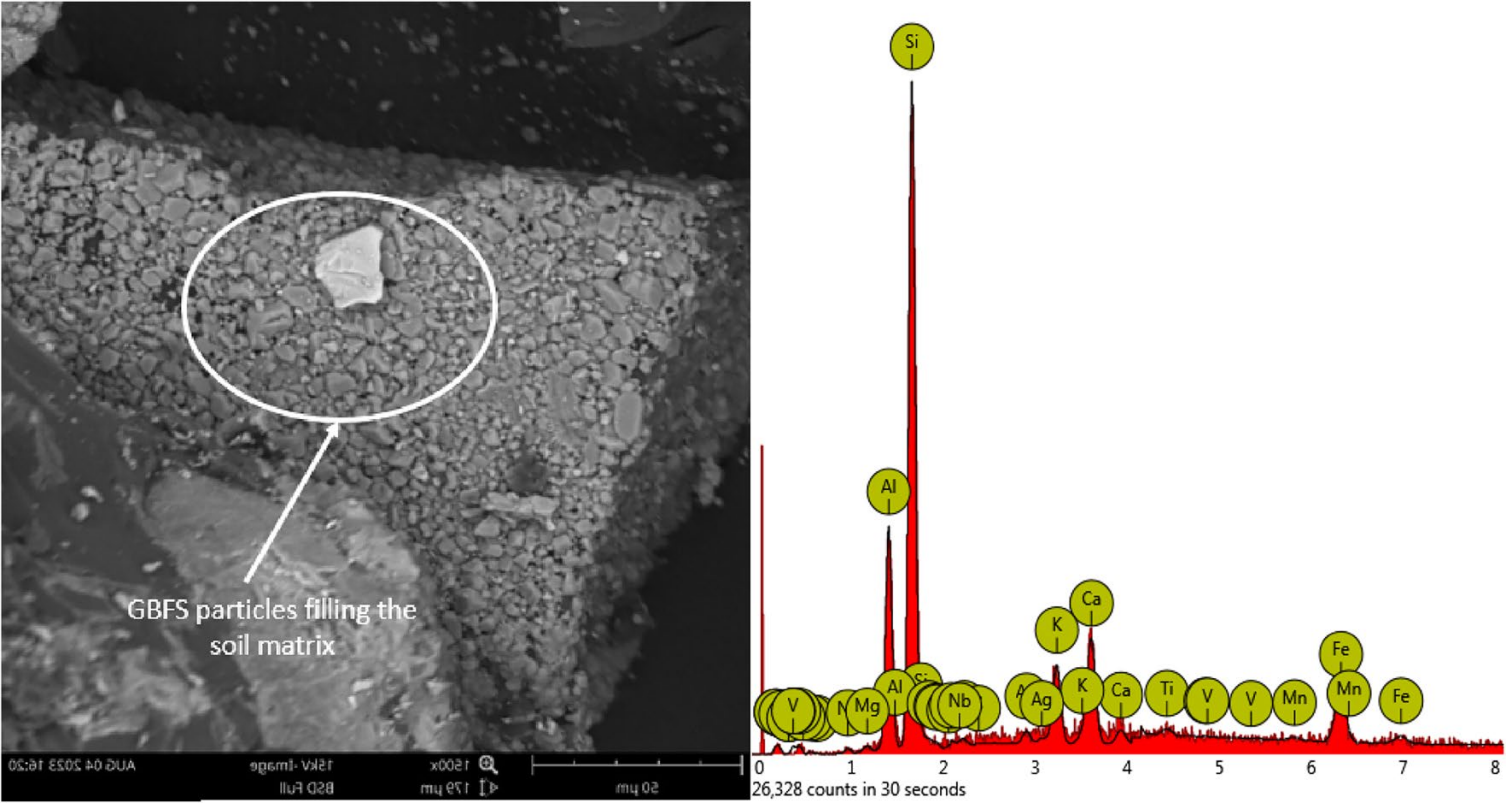
SEM–EDX of Bentonite and GBFS sample.

Figure 24 provides a quantitative scrutiny of the elemental concentrations and the micrograph of the 10% GBFS sample. Silicon (Si), prevailing predominantly in the mixture with an atomic concentration of 58.05, emanates from both the GBFS component and potentially other constituents. This substantial presence notably shapes the composite’s structural behaviour, playing a pivotal role in contributing to overall mechanical properties^65,66^. Similarly, Aluminium (Al) holds a significant atomic concentration of 12.76, underscoring its substantial involvement in the composite. Alumina, represented by Al_2_O_3_ with a stoichiometric weight concentration of 14.65, augments the material’s chemical composition, implying potential consequences for reactivity and strength attributes. Iron (Fe), accounting for 11.29 of the atomic composition, correspondingly appears as its oxide form, Fe_2_O_3_, with a stoichiometric weight concentration of 13.96. These iron-based components introduce ferrous compounds, potentially influencing both mechanical attributes and interactions with the surrounding environment. Calcium (Ca), comprising 5.60 of the atomic composition, assumes critical importance, with CaO (calcium oxide) contributing to a stoichiometric weight concentration of 9.04. The presence of calcium, commonly associated with GBFS, suggests contributions to stabilization and reactivity within the composite. Titanium (Ti), constituting 4.88 of the mixture, aligns with TiO_2_ (titanium dioxide) at a stoichiometric weight concentration of 4.66. The prevalence of titanium prompts considerations related to material durability, strength, and its potential impact on overall behaviour. Potassium (K), measuring 4.33 in atomic concentration, presents as K_2_O with a stoichiometric weight concentration of 2.96, potentially influencing chemical interactions within the composite material.

**Figure 25:**
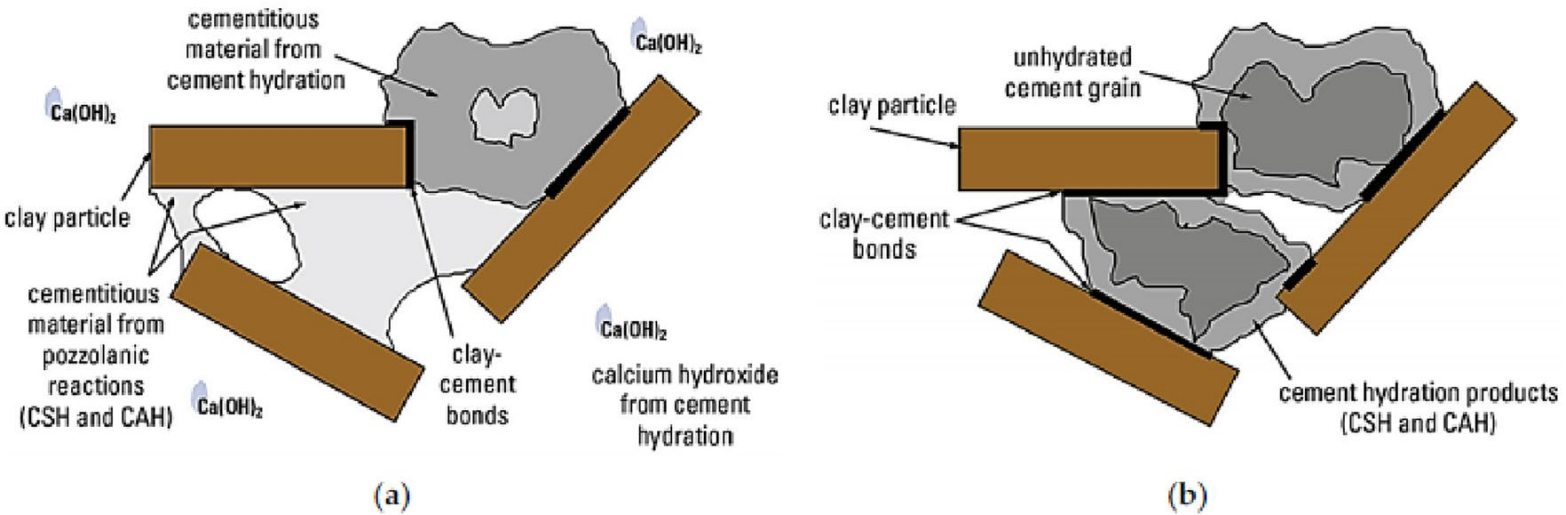
(**a**) Pozzolanic reaction between clay particles and binder (**b**) cementitious hydration activity between clay particles and binder^67^.

The Fig. 25 gives further illustration as regards the formation of cementitious materials due to the influence of the additives on the expansive soil.

## Conclusion

In this study, the compaction and compressibility characteristics of snail shell ash and ground-granulated blast-furnace slag -stabilized local bentonite for baseliner of landfill was assessed. The findings lead to the following conclusions.

- The untreated soil had a liquid limit of 65%, plastic limit of 35%, and plasticity index of 30%, with an optimum moisture content of 32% and a maximum dry density of 1423 kg/m^3^.
- Snail Shell Ash (SSA) contained 91.551 wt% CaO and trace elements, while Granulated Blast Furnace Slag (GBFS) had 53.023 wt% SiO2 and other components.
- Samples treated with 20% GBFS and 5% SSA showed the highest maximum dry density (1561 kg/m^3^) and an optimum moisture content of 13%, outperforming other mixtures.
- The 15% SSA-treated sample achieved the best strength enhancement, with unconfined compressive strength (UCS) reaching 272.61 kPa over 28 days, surpassing the 10% GBFS-treated sample with 229.95 kPa.
- The 15% SSA combination demonstrated the highest shear strength (49 kPa), elastic modulus (142 MPa), and favorable hydraulic conductivity (5.57 × 10^−8^ cm/s). Scanning Electron Microscopy (SEM–EDS) analysis of the 15% SSA mixture displayed substantial changes in composition, indicating its potential as a base liner within landfill systems. Overall, this sample exhibited the most robust mechanical properties and hydraulic conductivity, making it a promising option for landfill liner applications.

### Addressing the research gaps

The experimental investigation addressed several highlighted research gaps related to the compaction and compressibility characteristics of snail shell ash (SSA) and granulated blast furnace slag (GGBS) stabilized local bentonite for landfill baseliner applications. The research systematically blended different proportions of SSA and GGBS with local bentonite and testing various combinations to evaluate their impact on engineering properties.

- Settlement properties were assessed through consolidation tests, where specimens of the stabilized material were subjected to incremental loads over time, and the resulting settlements were measured. The results provide insights into the potential settlement behaviour of the blended material in landfill applications.
- Triaxial shear tests were conducted to evaluate the material’s resistance to shear stresses. Different combinations of SSA and GGBS blended with the soft soil were tested to assess their influence on shear strength.
- Hydraulic conductivity tests were performed to evaluate the material’s ability to resist the flow of water. Varying proportions of SSA and GGBS blended with the soft soil were examined to determine their impact on hydraulic conductivity.
- The sensitivity of unconfined compressive strength to various factors, such as stress conditions and material composition, were investigated through a series of tests. This would help identify the factors that most significantly affect the material’s strength.
- Compaction tests (standard Proctor) were conducted to evaluate the material’s compaction characteristics. Different blends of SSA and GGBS were tested to determine their influence on compaction properties.
- Scanning electron microscopy (SEM) coupled with energy-dispersive X-ray spectroscopy (EDS) were used to analyse the microstructure and mineralogical composition of the stabilized material. This analysis provides insights into the interaction between SSA, GGBS, and bentonite at the microscale.
- The obtained results were matched with EPA regulatory requirements which indicated enhanced properties for the SSA–GGBS blended soil compared to the unblended soil. Through these investigations, the study aimed to provide insights into the suitability and performance of the stabilized material for landfill baseliner applications.

## Data Availability

The datasets generated and analysed during the current study are available from the corresponding author on reasonable request.
